# Stress cross-response of the antioxidative system promoted by superimposed drought and cold conditions in *Coffea* spp.

**DOI:** 10.1371/journal.pone.0198694

**Published:** 2018-06-05

**Authors:** José C. Ramalho, Ana P. Rodrigues, Fernando C. Lidon, Luís M. C. Marques, A. Eduardo Leitão, Ana S. Fortunato, Isabel P. Pais, Maria J. Silva, Paula Scotti-Campos, António Lopes, F. H. Reboredo, Ana I. Ribeiro-Barros

**Affiliations:** 1 Plant-Environment Interactions & Biodiversity Lab (PlantStress&Biodiversity), Linking Landscape, Environment, Agriculture and Food Unit (LEAF), Dept. Recursos Naturais, Ambiente e Território (DRAT), Instituto Superior de Agronomia (ISA), Universidade de Lisboa (ULisboa), Oeiras, Portugal; 2 Unidade de Geobiociências, Geoengenharias e Geotecnologias (GeoBioTec), Faculdade de Ciências Tecnologia (FCT), Universidade NOVA de Lisboa (UNL), Caparica, Portugal; 3 Colóides Polimeros e Superficies, Instituto de Tecnologia Química e Biológica (ITQB), Universidade NOVA de Lisboa (UNL), Oeiras, Portugal; 4 Unid. Investigação em Biotecnologia e Recursos Genéticos, Instituto Nacional de Investigação Agrária e Veterinária, I.P. (INIAV), Oeiras, Portugal; Estacion Experimental del Zaidin, SPAIN

## Abstract

The understanding of acclimation strategies to low temperature and water availability is decisive to ensure coffee crop sustainability, since these environmental conditions determine the suitability of cultivation areas. In this context, the impacts of single and combined exposure to drought and cold were evaluated in three genotypes of the two major cropped species, *Coffea arabica* cv. Icatu, *Coffea canephora* cv. Apoatã, and the hybrid Obatã. Crucial traits of plant resilience to environmental stresses have been examined: photosynthesis, lipoperoxidation and the antioxidant response. Drought and/or cold promoted leaf dehydration, which was accompanied by stomatal and mesophyll limitations that impaired leaf C-assimilation in all genotypes. However, Icatu showed a lower impact upon stress exposure and a faster and complete photosynthetic recovery. Although lipoperoxidation was increased by drought (Icatu) and cold (all genotypes), it was greatly reduced by stress interaction, especially in Icatu. In fact, although the antioxidative system was reinforced under single drought and cold exposure (e.g., activity of enzymes as Cu,Zn-superoxide dismutase, ascorbate peroxidase, APX, glutathione reductase and catalase, CAT), the stronger increases were observed upon the simultaneous exposure to both stresses, which was accompanied with a transcriptional response of some genes, namely related to APX. Complementary, non-enzyme antioxidant molecules were promoted mostly by cold and the stress interaction, including α-tocopherol (in *C*. *arabica* plants), ascorbate (ASC), zeaxanthin, and phenolic compounds (all genotypes). In general, drought promoted antioxidant enzymes activity, whereas cold enhanced the synthesis of both enzyme and non-enzyme antioxidants, the latter likely related to a higher need of antioxidative capability when enzyme reactions were probably quite repressed by low temperature. Icatu showed the wider antioxidative capability, with the triggering of all studied antioxidative molecules by drought (except CAT), cold, and, particularly, stress interaction (except ASC), revealing a clear stress cross-tolerance. This justified the lower impacts on membrane lipoperoxidation and photosynthetic capacity under stress interaction conditions, related to a better ROS control. These findings are also relevant to coffee water management, showing that watering in the cold season should be largely avoided.

## Introduction

It is widely recognized that abiotic stresses, such as extreme temperatures, drought, or salinity, are major limiting factors to agriculture sustainability, more than halving average yields for major crop species [1]. Under field conditions, multiple stressors (*e*.*g*., extreme temperatures and water shortage) are frequently superimposed, with plants responding in ways not directly predictable from each single stress condition. In fact, stresses interaction can amplify or cancel the single stress responses on metabolism, mineral balance, and gene expression [2–4]. Moreover, responsive signaling pathways to abiotic stresses constitute an interconnected network that crosstalk at several levels [5,6], with each particular stress combination requiring a unique acclimation response [7].

In general, low positive temperatures (usually below 10°C) and water shortage affect photosynthesis, nutrient uptake, and crop yield, quality and post-harvest preservation [8,9]. Both stresses can affect virtually all photosynthetic components provoking, *e*.*g*., stomatal closure (thus, reducing net photosynthesis and sugar metabolism), changes on pigment complexes, reduction of photochemical efficiency and enzymes activity. Additionally, chilling reduces chemical reactions, and affects the lipid matrix of membranes, namely at the chloroplast level, further impairing thylakoid electron transport [8,10,11].

In plants, chloroplasts, mitochondria, and peroxisomes are major contributing sources of reactive oxygen species (ROS), due to several oxidative and electron transport reactions [12]. Within the chloroplast, PSI and PSII reaction centers are the major ROS generation sites [13], with 10 to 30% of thylakoid electron transport likely resulting in O_2_ photoreduction under adequate environmental conditions [14]. Moreover, ROS formation linked to photosynthesis is greatly affected by environmental stresses, particularly when photon energy capture exceeds that required for C-assimilation [13], in a process additionally affected by the reduction of photosynthate use by metabolic sinks [11]. This greatly increases the excitation energy transfer to Chl and O_2_, and an overproduction of highly reactive molecules, such as triplet and singlet state of Chl (^3^Chl* and ^1^Chl), singlet oxygen (^1^O_2_), and superoxide (O_2_●^-^) in PSI and PSII [13–15]. The O_2_●^-^ can further result in hydrogen peroxide (H_2_O_2_), and thereafter in hydroxyl radical (●OH) [16]. Collectively, these reactive species can promote lipoperoxidation, bleaching of pigments (*e*.*g*., P_680_), protein oxidation (*e*.*g*., D1), photosystems and enzymes inactivation, and DNA degradation [14,17,18]. Therefore, the upregulation of scavenging/detoxifying mechanisms that control the production and presence of highly reactive molecules is crucial for plant stress tolerance, namely to cold and drought [19–21]. This control may be achieved through the dissipation of energy excess (*e*.*g*., pigments, pseudocyclic electron transport, photorespiration), by overexpressing antioxidant enzymes, and by the action of non-enzymatic metabolites, such as hydrophilic (ascorbate, ASC, and glutathione, GSH), lipophilic (*e*.*g*., zeaxanthin, ZEA, β-carotene and α-tocopherol, TOC), and phenolic compounds [14,15,18,19,21]. Among the most important chloroplast antioxidative enzymes are Cu,Zn-superoxide dismutase (Cu,Zn-SOD), ascorbate peroxidase (APX), and glutathione reductase (GR), which are frequently complemented with the extra chloroplast action of catalase (CAT), when H_2_O_2_ diffuses to out of chloroplast. SOD dismutes superoxide radical (O_2_●^-^) into H_2_O_2_, but this reactive molecule is also highly toxic due to its own action, and because it can be transformed to hydroxyl radical (●OH) through the Haber-Weiss reaction. Therefore, H_2_O_2_ must be quickly scavenged into water by APX (together with ASC) and CAT enzymes. ASC is afterwards regenerated by monodehydroascorbate reductase (MDAHR), and dehydroascorbate reductase (DAHR), involving also GSH (regenerated by GR), and ZEA [14,21]. Several non-enzyme molecules contribute as well to ROS control. ASC and TOC scavenge ^1^O_2_, O_2_●^-^, ●OH and lipid peroxyl radicals non-enzymatically [16,21–23]. Moreover, phenolic compounds have been reported to be more effective ROS scavengers *in vitro* than TOC and ASC, with a major role in plant adaptation to biotic and abiotic stresses in some species [15,24,25]. Among them, caffeoylquinic acids (CQAs) are often produced in response to oxidative stress conditions (namely, induced by cold), scavenging free radicals as O_2_●^-^ [15], and preventing lipid peroxidation [26]. Additionally, ZEA scavenges ^1^O_2_, and acts through thermal dissipation of excess of light energy, reducing the formation of highly reactive molecules of Chl (^3^Chl* and ^1^Chl*), and protecting LHCs and membrane lipids against photooxidation [10,27–29].

Coffee is one of the world’s most important agricultural commodities, supporting the economy of many countries in the tropical region. Coffee bean production largely results from the cultivation of the species *C*. *arabica* L. and *C*. *canephora* Pierre ex A. Froehner [30], generating a global income around USD 173,400 million [31]. Moreover, the livelihoods of *ca*. 25 million farmers, mainly smallholders, depend on this highly labor-intensive crop [32], and *ca*. 100 million people are involved in the entire chain of value [33]. The demand for coffee beans is steadily increasing, but this crop could be endangered in several regions by the ongoing and future global climate changes, particularly as regards drought and unfavourable temperatures, which are the major climatic determinants for the suitability of coffee growing areas [30]. Recent reports showed positive impacts of elevated air [CO_2_] regarding the mitigation of heat impacts at leaf physiological and mineral levels [3,34,35], as well as bean quality [36] of coffee. However, it is known that under the actual climate conditions this crop is being progressively affected, showing substantial production and quality losses, associated with periods of extreme droughts combined with unfavourable temperatures [37–40]. In fact, photosynthesis is strongly affected and productivity can be reduced up to 80% in marginal regions in very dry years [30,41]. With regard to sub-optimal temperatures, monthly averages below 15–16 ^o^C negatively impacts coffee plant growth and yield [30], below 18 ^o^C C-assimilation is significantly reduced [42], and chilling causes non-readily reversible impairments on the photosynthetic machinery [43–45]. Cold have also implications in coffee fruit/bean development, chemical composition, and quality [46]. Still, relevant cold tolerance has been reported associated to the ability to maintain membrane stability [47,48], and reinforced antioxidative capability [49–51].

The superimposition of cold and drought limitations is a naturally occurring situation in tropical regions, but their mode of interaction, and the underlying response mechanisms, remains poorly understood as regards important crops [7]. Recognizing the crucial role of antioxidative mechanisms in the coffee acclimation to cold [42,49], drought [41], high irradiance and nitrogen starvation [52,53], we report for the first time the basis of coffee plant response to the combined exposure to drought and cold, including their aftereffects, in genotypes from the two main cultivated *Coffea* species.

## Material and methods

### Plant material and growth conditions

For the experiments were used plants from *C*. *arabica* L. cv. Icatu Vermelho (IAC 4045, an introgressed variety from *C*. *canephora* Pierre ex A. Froehner, resulting from a cross of *C*. *canephora* and *C*. *arabica* cv. Bourbon Vermelho, then further crossed to *C*. *arabica* cv. Mundo Novo), and Obatã Vermelho (IAC 1669–20, resulting from the crossing of *C*. *arabica* cv. Villa Sarchi x Timor hybrid, then further crossed to *C*. *arabica* cv. Catuaí Vermelho), and *C*. *canephora* Pierre ex A. Froehner cv. Apoatã (IAC 3598–3), thus, representing the two main producing species. These genotypes have agronomic relevance, since Icatu and Obatã are improved and widely cropped cultivars, whereas Apoatã is frequently used in breeding programs for drought tolerance, and as rootstock against nematodes. Plants were grown in 16 L pots under greenhouse conditions, watered when needed (every 2 days in spring-summer and once a week in autumn-winter), and fertilized exactly as described in [54]. With 1.5 years of age, plants with similar size as regards the canopy size were then transferred into walk-in growth chambers (EHHF 10000, ARALAB, Portugal), and maintained under controlled environmental conditions of temperature (25/20°C, day/night), RH (70%), irradiance at the upper third part of plant canopy (750–850 μmolQ m^-2^ s^-1^), photoperiod (12 h) and air [CO_2_] (390 μL L^-1^), for 3 months to allow the development of new leaves and a complete plant acclimation to these stable environmental conditions (see S1 Fig). Determinations were carried out using the 2 top pairs of newly matured leaves from each branch, from the upper third part of the plant. For biochemical evaluations, leaf material was collected after *ca*. 2 h of illumination from 4 to 8 plants of each genotype and treatment, flash frozen in liquid N_2_ and kept at -80°C until analysis. Leaf tissue extractions were performed using an ice-cold mortar and pestle, as well as cold homogenizing solutions. Whenever possible, all analyses were performed on the same leaves.

### Imposition of drought and cold treatments

Drought and low temperature were imposed by gradual decrease of irrigation/temperature, as they normally occur in nature, in order to allow the plants to express eventual acclimation ability. Water availability levels were firstly established (in 15 plants per treatment), under adequate temperature (25/20°C, day/night), corresponding to control well-watered (WW); mild drought (MD), and severe drought (SD) conditions, representing *ca*. 80, 35 and 10% of maximal water availability in pots. These conditions were gradually imposed along two weeks, through a partial reposition of water lost in each pot, until stability of predawn leaf relative water content (RWC) and water potential (Ψ_w_) values. Such water availability conditions were thereafter kept for another week before the onset of cold conditions, as well as along the entire exposure to low temperature and cold recovery periods (see below) by adding the amount of water loss by the pot, as evaluated every two days by pot weighting, confirmed by leaf RWC and Ψ_w_ measurements, and by visual evaluation of hydration/wilting status throughout the entire experiment. Finally, 41 days after the establishment of water levels, plants were re-watered, and followed along a seven day drought recovery period (7x Rec Drought).

Cold treatment started one week after the stabilized water availability conditions have been achieved. The plants were then submitted to a gradual cold exposure and a recovery thereafter, exactly as previously described [45,51]. Briefly, plants were successively exposed to 1) a gradual temperature decrease (0.5 ^o^C per day) from 25/20 ^o^C to 13/8 ^o^C, over 24 days, to allow the expression of acclimation ability, 2) a 3 days chilling cycle (3x13/4 ^o^C), where 4 ^o^C were applied during the night and in the first 4 h of the morning (thus, with light), followed by a rise up to 13 ^o^C, throughout the rest of the diurnal period, 3) a rewarming period of 7 days (7x Rec Cold), with the first day after chilling at 20/15 ^o^C and the rest at 25/20 ^o^C, in order to allow recovery from cold conditions. Only then the droughted plants were fully watered and allowed to recover to another period of 7 days (7x Rec Drought).

The total experiment took *ca*. 62 days since the beginning of the setting of water availability levels (S1 Fig).

### Water status characterization and monitoring

Leaf relative water content (RWC) measurements were performed as described in [55], optimized for *Coffea* spp., using eight foliar discs of 0.5 cm^2^ each, punched from the same leaves used for water potential determinations. RWC values (%) were calculated as = [(FW-DW)/(TW-DW)]x100, where FW represents the fresh weight determined immediately after cutting the discs, TW is the turgid weight obtained after overnight rehydration of the discs in a humid chamber at *ca*. 20°C, and DW is the dry weight obtained after drying the discs at 80°C for 48 h.

Leaf water potential (Ψ_w_) was determined immediately after leaf excision from the plant, using a pressure chamber [56].

Both RWC and Ψ_w_ measurements were performed at predawn on 4–5 replicates per treatment, every two days, but are presented only the data at major data collection points (considering temperature decrease, as well as cold and drought recoveries).

### Leaf gas exchanges

Leaf gas exchanges were determined following [45]. Briefly, net photosynthesis was evaluated on 5–8 plants/treatment, under steady-state conditions after *ca*. 2 h of light exposure, using a CO_2_/H_2_O open system portable IRGA (CIRAS I, PP Systems, USA).

Measurements of O_2_ evolution expressing photosynthetic capacity, *A*_max_, were performed in leaf discs (1.86 cm^2^) under irradiance (PPFD 800–1000 μmol m^-2^ s^-1^) and CO_2_ (*ca*. 7%) saturating conditions, at 25°C, in a Clark-type leaf-disc O_2_ electrode (LD2/2, Hansatech, UK). Saturating PPFD was provided by a Björkman lamp (Hansatech).

### Lipid peroxidation evaluation

To evaluate lipid peroxidation level of leaf cell membranes, the thiobarbituric acid (TBA) test, which determines malondialdehyde (MDA) as a final product of lipid peroxidation, was performed according to [57], using 200 mg FW leaf samples. Quantification of MDA-TBA complex (red pigment) was obtained using the Abs_532nm_ value, subtracted from the non-specific Abs_600nm_, and an extinction coefficient of 155 mM^-1^ cm^-1^.

### Maximal cellular activity of antioxidative enzymes

#### Enzymes extraction

Procedures were performed in four replicates of freshly cut pooled samples of 100 mg FW leaf material (six plants per treatment), in 1 mL of ice cold buffer (4°C), as globally described in [58], with minor modifications to coffee leaves, including the addition of 1% PVPP to each sample in the homogenization.

For determination of maximal apparent activities of superoxide dismutase (SOD; EC 1.15.1.1) and glutathione reductase (GR; EC 1.6.4.2), leaf samples were homogenized in 100 mM sodium phosphate buffer (pH 7.8), containing 1.0% Triton X-100, 10% glycerol, 10 mM β-mercaptoetanol, 2 mM DTT, 2% “cOmplete-protease inhibitor cocktail” (ref. 04693116001, Roche), and 1% soluble PVP. The homogenate was centrifuged (10,000 *g*, 15 min, 4°C), using the supernatant to evaluate SOD and GR activities.

Ascorbate peroxidase (APX; EC 1.11.1.11) was extracted by homogenizing leaf samples in 100 mM sodium phosphate buffer (pH 7.8), containing 1.0% Triton X-100, 10% glycerol, 10 mM β-mercaptoetanol, 2 mM DTT, 2% “cOmplete-protease inhibitor cocktail”, 1% soluble PVP, and 2.0 mM ascorbic acid (ASC). The homogenate was centrifuged (10,000 *g*, 20 min, 4°C), using the supernatant to evaluate APX activity.

For catalase (CAT; EC 1.11.1.6) activity leaf tissue was homogenized in 1 mL of 100 mM sodium phosphate solution (pH 7.0), containing 1.0% Triton X-100, 10% glycerol, 10 mM β-mercaptoetanol, 2 mM DTT, 2% “cOomplete-protease inhibitor cocktail”, and 1% soluble PVP. The homogenate was centrifuged (10,000 *g*, 20 min, 4°C), using the supernatant to evaluate CAT activity.

#### Cellular activity assays

SOD activity assay was based on [59]. The enzyme reaction mixture contained 1.3 mM riboflavin, 13 mM methionine, 63 mM nitro blue tetrazolium (NBT) in 0.1 M phosphate buffer (pH 7.8), and 50 μL of the enzyme extract in a final volume of 3 mL. Glass test tubes containing the mixture were immersed in a bath at 25°C and illuminated for 15 min before readings at Abs_560nm_. One unit of SOD was defined as the enzyme activity which inhibited the photoreduction of NBT to blue formazan by 50%.

APX activity assay was based on [60]. The enzyme reaction mixture contained 0.5 mM ascorbate and 0.1 mM H_2_O_2_ in 50 mM phosphate buffer (pH 7.0) and 200 μL of the enzyme extract in a total volume of 1 mL. Activity was determined through H_2_O_2_-dependent oxidation of ascorbate (at Abs_290nm_), using an extinction coefficient of 2.8 mM^-1^ cm^-1^ for calculations.

GR activity assay reaction mixture contained 50 mM NADPH, 10 mM oxidized glutathione (GSSG), 3 mM MgCl_2_ in 0.1 M sodium phosphate buffer (pH 7.8), and 50 μL of enzyme extract in a total volume of 400 μL. GR activity was evaluated using the Abs_340nm_ decrease, corresponding to the NADPH oxidation rate [61].

CAT activity was measured according to [62]. The enzyme assay reaction mixture contained 0.1 mM H_2_O_2_ in 50 mM sodium phosphate buffer (pH 7.0) and 200 μL of the enzyme extract in a total volume of 3 mL. Activity was estimated based on the Abs_240nm_ decrease, related to H_2_O_2_ consumption. For calculation a standard curve with known H_2_O_2_ concentrations was performed.

All activity assays were performed at a stabilized temperature of 25°C.

The soluble protein content was determined according to [63], with bovine serum albumin used as a standard.

### Non-enzymatic antioxidants evaluation

#### Leaf carotenoids

Pigments were assessed from four leaf discs (each 0.5 cm^2^), which were cut after 1.5-2h of illumination, flash frozen in liquid nitrogen and stored at -80 ^o^C until analysis. The leaf tissue homogenization, and the subsequent reversed-phase HPLC analysis were performed as in [35], using an end-capped, C_18_, 5 μm Spherisorb ODS-2 column (250 x 4.6 mm, Waters, USA). Detection was performed at Abs_440nm_ in a HPLC system (Beckman, System Gold, USA) coupled to a diode-array (DAD Mod. 168, Beckman) detector. Identification and quantification of each pigment were performed with specific standards. The de-epoxidation state, involving the xanthophyll cycle components zeaxanthin (ZEA), antheraxantin (ANT) and violaxanthin (VIOL), was calculated as [DEPS = (ZEA+0.5ANT)/(VIOL+ANT+ZEA)].

### Ascorbate (vitamin C)

Determinations followed [64], with minor modifications for coffee leaves [49]. Briefly, 100 mg FW leaf samples were homogenized in 2 mL of a solution of 3% (w/v) meta-phosphoric acid and 4% (v/v) glacial acetic acid, left for 15 min with agitation and submitted to ultrasounds (5 min). The samples were then centrifuged (10,000 *g*, 5 min, 4 ^o^C) and filtered (PVDF, 0.45 μm) prior to a reversed-phase HPLC analysis, similar to that used for leaf carotenoids. The elution of a 20 μL sample aliquot was performed with H_2_O at pH 2.2 (addition of H_2_SO_4_), for 15 min, with a 0.4 mL min^-1^ flow rate, and detection at Abs_254nm_. ASC was quantified using a specific standard.

#### *α*-tocopherol (vitamin E)

Determinations were based in [65] and [23], with some changes for coffee leaves [49]. Briefly, 200 mg FW leaf tissue was homogenized in 3 mL of methanol, containing 0.24 mM of citric acid and 0.28 mM of isoascorbic acid, submitted to ultrasounds (5 min) and centrifuged (10,000 *g*, 5 min, 3°C). The supernatant was collected and the pellet was re-extracted, repeating the procedure twice. The supernatants were then combined, dried under vacuum, and the residue was re-suspended in 3 mL of acetonitrile, centrifuged (10,000 *g*, 3 min, 3°C) and filtered (PVDF, 0.45 μm), prior to a reversed-phase HPLC analysis, similar to that performed to ascorbate, except that a fluorescence detector (Jasco, FP1520, Japan, at 295 nm for excitation and 325 nm for detection) and methanol as eluent with a flow rate of 1 mL min^-1^, were used. TOC quantification was performed with a specific standard.

#### Total phenolic content

Total phenolic content was determined according to Folin-Ciocalteu method [66]. Briefly, 100 mg FW leaf samples were homogenized in 5 mL of a solution of 70% (v/v) methanol for 30 min under vigorous shaking (Variomag®Poly15, Thermo Fisher Scientific, USA) and filtered (PVDF, 0.45 μm). Thereafter, 20 μL of the extract was added to 1.48 mL of distilled water and oxidized with 100 μL of Folin-Ciocalteu reagent (Sigma-Aldrich). The reaction was neutralized with 300 μL of sodium carbonate and samples were then submitted to 30 min of incubation at 40°C. The Abs_765nm_ was measured using a Genesys 10 UV spectrophotometer (Thermo Spectronic, New York, USA). Results were expressed as gallic acid equivalent (mg GAE/g leaves extract dry weight).

#### Chlorogenic acid

Determinations followed [49]. Briefly, 400 mg FW leaf samples were homogenized in 5 mL methanol (with 1% HCl). After centrifugation (10,000 *g*, 10 min, 4°C) the supernatant was filtered (PVDF, 0.45 mm) prior to a reversed-phase HPLC analysis, similar to that performed to ascorbate. The elution of a 20 μL injection was performed at 23–24°C, over 30 min, with a 1 mL min^-1^ flow rate, using a linear gradient from 20 to 70% methanol (with1%HCl), in phosphoric acid (10 mM, pH 2.5). For Abs_325nm_ detection a diode-array detector (mod.168, Beckman) was used. Identification and quantification were performed using 5-caffeoylquinic acid (5-CQA) solutions with known concentrations.

#### Expression studies of selected genes

Total RNA was isolated and quantified as described in [67]. One microgram of DNA-free total RNA was used to synthesize first-strand cDNAs using oligo-(dT)18 primers and the SuperScriptII first-strand synthesis system (Invitrogen, USA).

Genes related to proteins involved in the antioxidative response were selected for the expression studies. Based on available gene sequences libraries [68,69] primers were designed using Primer3 [70], and checked using Oligo Calculator [71] (Table 1). To determine the specificity of each primer pairs, melting/dissociation curve analysis was performed following the RT-qPCR experiment. A single peak in the obtained melting curve confirmed the specificity of the amplicon. No signal was detected in the negative controls. Relative gene expression must be calculated after normalization with multiple reference genes [72]. For that purpose ubiquitin (*UBQ10*), glyceraldehyde 3-phosphate dehydrogenase (*GAPDH*), and Cyclophilin (*Cycl*) were used, as the most reliable stable reference genes for coffee under the actual experimental conditions [67].

**Table 1:** Selected genes used for real-time qPCR studies. Selected genes related to the oxidative stress control, homologies, primer sequences, access number on NCBI GenBank and amplicon size (bp).

Gene Symbol	Primer Sequence (5'-3')	Gene Description	NCBI GenBank Access Number	Amplicon Size (bp)
UBQ2*	F: GATGATACTTGGCCCTGCAC	Ubiquitin-conjugating enzyme E2	GR984245	142
	R: CCTTCCCAGCTTGTCAATGT			
*APXc*	F: GATTGCCTTTTGCTGTCTGATG	Putative cytosolic ascorbate peroxidase (cAPX)	JQ013438.1	132
	R: CGGGAATATGAACGACCACATA			
*APXm*	F: GAACTGGGTTTTACTCCACATTCC	Membrane-bound ascorbate peroxidase (mAPX) mRNA	JQ013439.1	119
	R: CAAGTAACTGAGAACCACAACTGC			
*APXt+s*	F: AGGGCAGAATATGAAGGATTGG	Stromatic ascorbate peroxidase (sAPX) mRNA	JQ013441.1	112
	R: CCAAGCAAGGATGTCAAAATAGCC			
*PX4*	F: CCAAGTTCTTATGAGCGACAACAC	Putative class III peroxidase (POX4)	JQ013435.1	106
	R: TGCCCATCTTTACCATTGACAC			
*VDE2*	F: GGGTTCAAAATGCACAAGACTG	Violaxanthin de-epoxidase	DQ234768.1	86
	R: CCCTCTTTTACCTCAGGCATTG			

* Used to check for DNA contamination in RNA samples and positive control for cDNA synthesis.

SE of normalized expression levels were calculated according to the error propagation rules, according to the formula: SE = GI_norm_×((SD_NF_/NF)^2^+(SD_GI_/GI)^2^)^0.5^/m^0.5^, where *GI*_*norm*_ is the normalized relative expression of the gene of interest, *SD*_*NF*_ is the standard deviation of the normalization factor, *NF* is the normalization factor, *SD*_*GI*_ is the standard deviation of the quantities of the gene of interest, *GI* is the quantity calculated for the gene of interest, and *m* is the number of replicates [72].

### Statistical data analysis

The various compounds and parameters were analyzed using two-way ANOVAs (P ≤ 0.05) to evaluate the differences between temperature and water availability treatments, as well as their interaction, followed by a Tukey test for mean comparisons for a 95% confidence level. Each ANOVA was performed independently for each of the studied genotypes. Overall, the water availability x temperature interaction for most parameters was significant (Table 2). To the sake of simplicity we also did not consider the comparison between genotypes within each water and temperature treatments.

**Table 2:** ANOVA results regarding the impact of temperature, water availability, and their interaction (P ≤ 0.05), independently for each of the studied genotypes. ANOVA results (P ≤ 0.05) for the leaf studied parameters are: relative water content, RWC; water potential, Ψ_W_; net photosynthesis, P_n_; photosynthetic capacity, A_max_; malondialdehyde content, MDA; maximal activities of Cu,Zn-superoxide dismutase, Cu,Zn-SOD, ascorbate peroxidase, APX, glutathione reductase, GR, and catalase, CAT; *α*-tocopherol content, TOC; ascorbate content, ASC; zeaxanthin content, ZEA; sum of the xanthophylls violaxanthin, antheraxanthin and zeaxanthin content, V+A+Z; xanthophylls de-epoxidation state, DEPS; total phenolic content, Total Phenols.

Variables	Temperature			Water Availability			Interaction		
	Apoatã	Icatu	Obatã	Apoatã	Icatu	Obatã	Apoatã	Icatu	Obatã
RWC	*	*	*	*	*	*	*	*	*
Ψ_W_	*	*	*	*	*	*	*	*	*
P_n_	*	*	*	*	*	*	NS	*	*
A_max_	*	*	*	NS	*	*	NS	*	*
MDA	*	*	*	*	*	NS	*	*	NS
Cu,Zn-SOD	*	*	*	*	*	*	*	*	*
APX	*	*	*	*	*	*	NS	*	*
GR	*	*	*	*	*	*	*	*	*
CAT	*	*	*	*	NS	*	*	NS	*
TOC	*	*	*	*	*	NS	*	NS	NS
ASC	*	*	*	*	*	*	*	NS	NS
ZEA	*	*	*	*	*	*	*	*	*
V+A+Z	*	*	*	*	NS	*	*	NS	*
DEPS	*	*	*	*	*	*	*	*	*
Total Phenols	*	*	*	*	*	*	*	*	*
5-CQA	*	*	*	*	*	*	*	*	*

*—significant; NS–non-significant.

The relative expression ratio of each target gene was computed based on its real-time PCR efficiency and the crossing point (CP) difference of a target sample *versus* control (25/20°C, WW) within each genotype. Data analysis was performed with Relative Expression Software Tool [73]. A 95% confidence level was adopted for all tests.

## Results

### Stress imposition and characterization of water status

The single imposition of water shortage under control temperature (25/20°C) led to significant differences between the well-watered (WW) and severe droughted (SD) plants in all genotypes, as regards predawn values of RWC and Ψ_w_, while mild droughted (MD) plants showed intermediate values (Table 3). Although in Icatu the RWC and Ψ_w_ values of MD and SD plants were lower than in the other two genotypes, all three genotypes were effectively submitted to three water availability regimes from the beginning of the experiment. This successful establishment and maintenance of water availability unquestionably allowed the evaluation of single and combined impacts of water deficit and low temperature for these genotypes.

**Table 3:** Values of leaf relative water content (RWC, %) and water potential (Ψ_W_, MPa). Values were obtained at predawn along the entire experiment for Apoatã, Icatu, and Obatã genotypes, under well-watered (WW), mild drought (MD) and severe drought (SD) conditions, and submitted to temperature control conditions (25/20 ^o^C), during the gradual temperature decrease (18/13 ^o^C), at the end of the acclimation period (13/8 ^o^C), after 3 chilling cycles (3x13/4 ^o^C), after 7 days under rewarming conditions (7x Rec Cold), and after a further 7 days period under rewatering conditions (7x Rec Drought).

Genotype	Treatment	Temperature (day/night)																							
		25/20°C				18/13°C				13/8°C				3x13/4°C				7x Rec Cold				7x Rec Drought			
		**Predawn RWC (%)**																							
	**WW**	92.1	±	3.3	aA	94.5	±	1.4	aA	79.2	±	6.8	bcAB	73.0	±	5.4	cA	90.9	±	0.5	abA	91.8	±	0.7	aA
**Apoatã**	**MD**	88.4	±	2.8	abAB	85.0	±	1.5	abAB	83.8	±	2.8	abA	77.2	±	4.9	bA	82.5	±	4.1	abA	89.4	±	2.7	aA
	**SD**	82.9	±	3.1	abB	78.2	±	6.5	abcB	72.0	±	5.4	bcB	69.1	±	6.5	cA	82.2	±	3.1	abA	84.5	±	2.3	aA
	**WW**	92.3	±	1.8	abA	88.9	±	2.2	abA	87.3	±	2.1	abA	81.1	±	3.2	bA	88.3	±	3.4	abA	92.4	±	0.9	aA
**Icatu**	**MD**	79.7	±	2.6	abB	80.7	±	1.8	abB	77.3	±	2.0	bB	73.8	±	2.0	bAB	78.2	±	3.0	bB	89.1	±	1.0	aA
	**SD**	69.5	±	3.4	bC	63.0	±	2.7	cC	71.0	±	1.1	bB	72.3	±	0.8	bB	64.4	±	3.8	bcC	89.0	±	0.9	aA
	**WW**	91.2	±	1.1	aA	89.0	±	1.6	aA	85.2	±	2.2	aA	83.5	±	2.3	aA	86.3	±	2.6	aA	87.3	±	1.5	aA
**Obatã**	**MD**	86.5	±	1.1	aAB	83.8	±	2.4	aAB	81.2	±	1.4	aAB	82.6	±	2.1	aA	79.4	±	1.5	aAB	86.7	±	0.7	aA
	**SD**	82.8	±	2.7	abB	74.6	±	4.9	bB	74.0	±	2.9	bB	79.2	±	3.0	aA	77.3	±	1.6	abB	85.6	±	1.6	aA
		**Predawn Ψ**_**W**_ **(MPa)**																							
	**WW**	-0.42	±	0.06	aA	-0.54	±	0.06	aA	-0.66	±	0.10	aA	-0.63	±	0.09	aA	-0.41	±	0.05	aA	-0.42	±	0.06	aA
**Apoatã**	**MD**	-0.72	±	0.07	abAB	-0.88	±	0.05	abA	-1.22	±	0.14	bAB	-0.84	±	0.09	abA	-0.83	±	0.28	abAB	-0.43	±	0.03	aA
	**SD**	-1.02	±	0.24	abB	-1.51	±	0.21	bB	-1.61	±	0.31	bB	-1.06	±	0.03	abA	-1.32	±	0.45	bB	-0.47	±	0.04	aA
	**WW**	-0.40	±	0.04	aA	-0.56	±	0.07	aA	-0.85	±	0.12	aA	-0.49	±	0.12	aA	-0.37	±	0.04	aA	-0.37	±	0.03	aA
**Icatu**	**MD**	-1.11	±	0.04	bB	-1.46	±	0.22	bcB	-1.99	±	0.10	cdB	-2.22	±	0.18	dB	-2.16	±	0.09	dB	-0.40	±	0.05	aA
	**SD**	-2.86	±	0.07	bC	-3.13	±	0.39	bC	-2.96	±	0.28	bC	-2.95	±	0.27	bC	-3.24	±	0.36	bC	-0.42	±	0.03	aA
	**WW**	-0.46	±	0.05	aA	-0.56	±	0.06	aA	-0.81	±	0.09	aA	-0.55	±	0.04	aA	-0.37	±	0.04	aA	-0.45	±	0.06	aA
**Obatã**	**MD**	-0.86	±	0.12	abAB	-0.98	±	0.21	abA	-1.83	±	0.14	cB	-1.31	±	0.15	bcB	-1.35	±	0.24	bcB	-0.45	±	0.03	aA
	**SD**	-1.33	±	0.21	bB	-2.10	±	0.09	cB	-2.49	±	0.12	cC	-2.01	±	0.09	cC	-2.12	±	0.32	cC	-0.56	±	0.06	aA

For each parameter, the mean values ± SE (n = 5) followed by different letters express significant differences between temperature treatments for the same water availability level (a, b, c), or between water treatments for each temperature treatment (A, B, C), always separately for each genotype.

Droughted plants also differed visually, with MD plants becoming wilted by the end of the diurnal period, whereas SD plants were permanently wilted along the diurnal period. This visual impact was not so drastic in Apoatã due to greater structural leaf rigidity.

Leaf dehydration was also promoted by the gradual cold imposition, as observed on the RWC value of WW plants of all genotypes by the end of the acclimation period (13/8°C) and/or after chilling exposure (3x13/4°C). This resulted in closer RWC values between the plants of the three water conditions under cold than at control temperature, without significant differences between water availability treatments in most cases after 4°C exposure. Such cold-promoted dehydration in WW plants was reverted to values close to control temperature after 7 recovery days (7x Rec Cold).

At 13/8°C the RWC values of SD plants were similar between genotypes, although with strong differences between their Ψ_w_ values. The same was observed when comparing Apoatã and Icatu after night chilling conditions. Notably, Icatu plants showed the lowest Ψ_w_ values from 13/8°C until 7x Rec Cold, both for MD (*ca*. -2 MPa) and SD (*ca*. -3 MPa), but recovered as much as Apoatã and Obatã plants 7 days after rewatering (7x Rec Drought).

**Fig 1:**
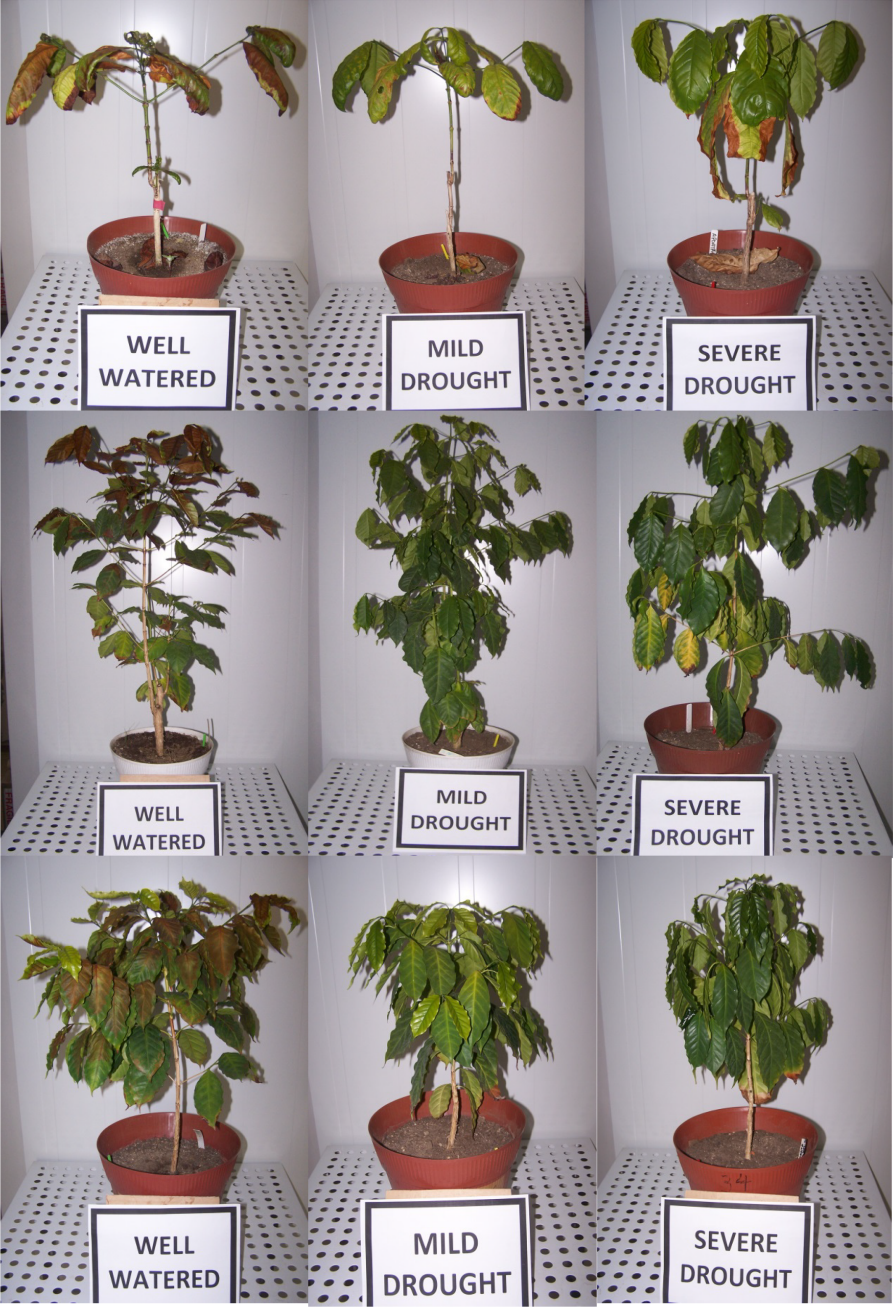
Visual cold impact at the leaf level. Impacts noted after 3 chilling cycles (3x13/4 ^o^C) exposure in Apoatã (upper), Icatu (middle), and Obatã (lower) genotypes, under well-watered, mild drought and severe drought conditions.

By the end of the experiment, plant visual evaluation revealed substantial differences of the stress impact between genotypes and treatments. Apoatã showed a greater leaf area loss, heavier in the SD treatment with leaves frequently becoming yellowish and necrotic, but also through shed of apparently normal green leaves (data not shown). On the other hand, although showing strong loss of leaf turgor (clear wilted look) Icatu MD and SD plants did not present any leaf loss or necrotic injury for the entire cold exposure period, contrary to Icatu WW plants that presented important leaf area loss, although in a lower extent than Obatã and, especially, Apoatã. Such visual impact, regarding a strong leaf necrosis and shed in WW plants of all genotypes, was evident after the exposure to 3 chilling cycles (3x13/4°C) (Fig 1).

### Photosynthetic parameters

**Fig 2:**
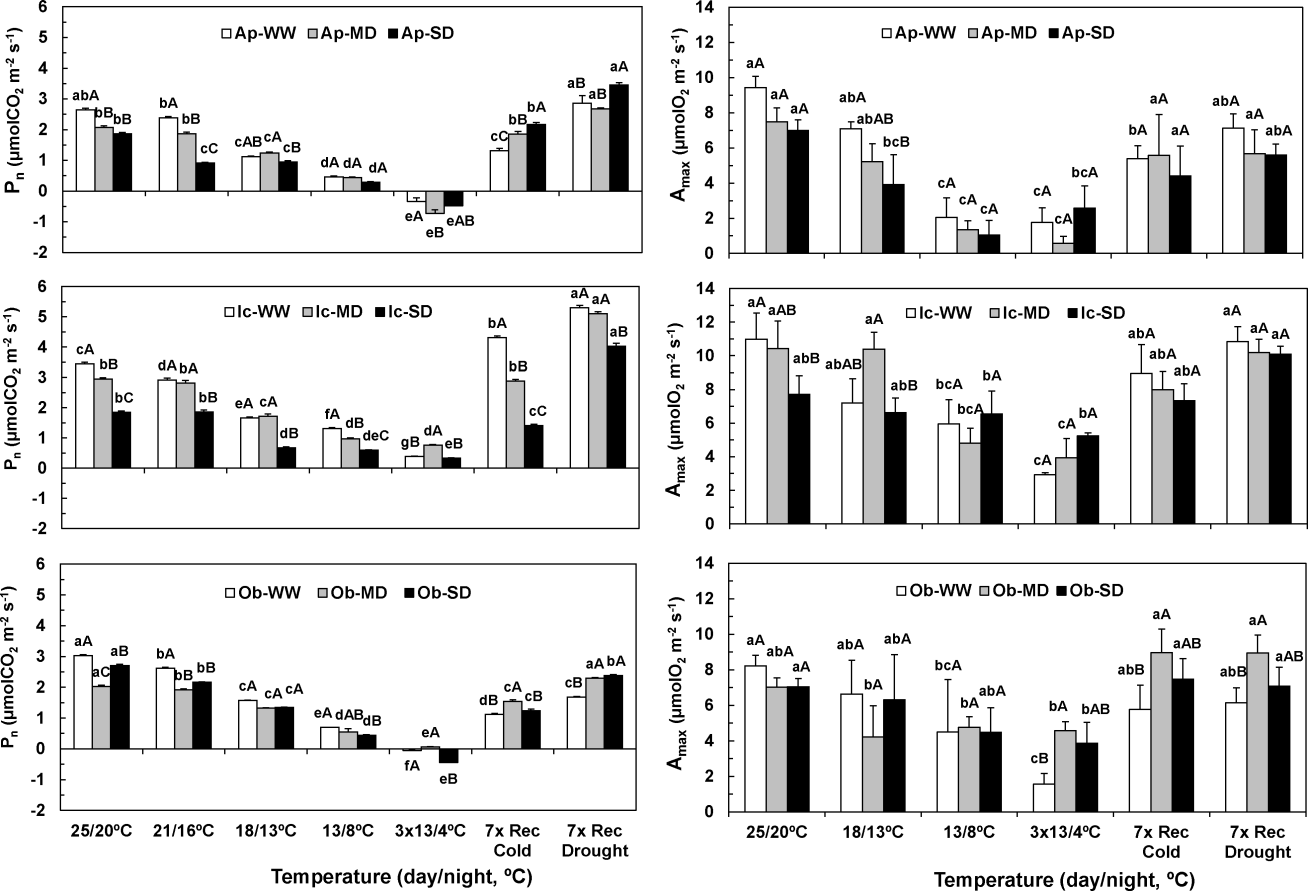
Impact at the leaf assimilation level. Changes in net photosynthesis (P_n_) (left) and photosynthetic capacity (A_max_) (right) values along the entire experiment for Apoatã (Ap), Icatu (Ic), and Obatã (Ob) genotypes, under well-watered (WW), mild drought (MD) and severe drought (SD) conditions, and submitted to temperature control conditions (25/20 ^o^C), during the gradual temperature decrease (18/13 ^o^C), at the end of the acclimation period (13/8 ^o^C), after 3 chilling cycles (3x13/4 ^o^C), after 7 days under rewarming conditions (7x Rec Cold), and after a further 7 days period under rewatering conditions (7x Rec Drought). For each parameter, the mean values ± SE (n = 5–8) followed by different letters express significant differences between temperature treatments for the same water availability level (a, b, c, d, e, f), or between water treatments for each temperature treatment (A, B, C), always separately for each genotype.

Water deficit alone provoked reductions in the assimilation rate (P_n_) under controlled temperature in all genotypes (Fig 2). SD conditions reduced P_n_ by 29%, 46% and 11%, respectively, for Apoatã, Icatu and Obatã, but in the latter genotype a reduction of 33% was observed in MD plants. Similarly, the photosynthetic capacity (A_max_) showed reductions of 26%, 30% and 14%, for the same genotype order (significant only for Icatu).

The single exposure to cold (WW plants) promoted P_n_ reductions, significantly from 21/16°C (Icatu and Obatã) or 18/13°C (Apoatã) onwards. Negligible values were found after chilling exposure in all WW plants, but strong differences between genotypes arose along cold recovery. In fact, with 7 days of cold recovery, Icatu showed values higher than control, whereas in Apoatã a total recovery was found only after 15 days, and in Obatã a 45% reduction was still present by the end of the experiment. For A_max_, a significant negative effect of cold on WW plants was observed at 13/8°C and chilling exposure. Still, at 7x Rec Cold such significant impact persisted in Apoatã and Obatã, but not in Icatu, the only genotype that showed a total A_max_ recovery by the end of the experiment.

With the imposition of water deficit previously to cold exposure, some differences were noted in the response to low temperatures, particularly in the recovery period. During temperature decrease, P_n_ values become closer among water treatments, but in most cases WW maintained higher values than SD plants until 13/8°C. After chilling exposure, only Icatu maintained positive P_n_ values, although quite low, between 10% (SD) and 22% (MD) of the WW value at 25/20°C. However, by 7x Rec Cold droughted Apoatã and Obatã plants recovered better than WW ones, an effect extended even after rewatering. In the case of Icatu, the WW recovered better by 7x Rec Cold, but MD plants showed already a strong recovery to a value similar of these plants at the beginning of the experiment, representing 84% of the control value even under water shortage conditions. After rewatering, Icatu WW and MD showed close values, nearly 50% above the initial control value. Even SD plants showed a 16% higher value than at 25/20°C and WW conditions.

As regards A_max_, the trends were somewhat different than in P_n_ in what concerns the impact of the combined stress imposition. In Icatu and Obatã the MD and SD plants kept close values to WW ones along the temperature decrease, but with a consistent tendency to higher values after 4°C exposure. Afterwards a prompt cold recovery was found even in the MD and SD plants, particularly in Icatu plants that showed values representing more than 90% of those obtained at 25/20°C. A complete A_max_ drought recovery was observed by the end of the experiment. In Obatã, MD plants (and partially SD) recovered faster to cold and drought stresses, reaching values higher than its control (25/20°C, WW). On the other hand, Apoatã showed a tendency to higher impact on A_max_ in droughted plants until 13/8°C, but without differences from this point forward. Contrary to the complete P_n_ recovery, this genotype showed the worst A_max_ recover by the end of the experiment, with values representing 75% (WW), 60% (MD) and 59% (SD) of the initial control value.

### Lipoperoxidation assessment

**Fig 3:**
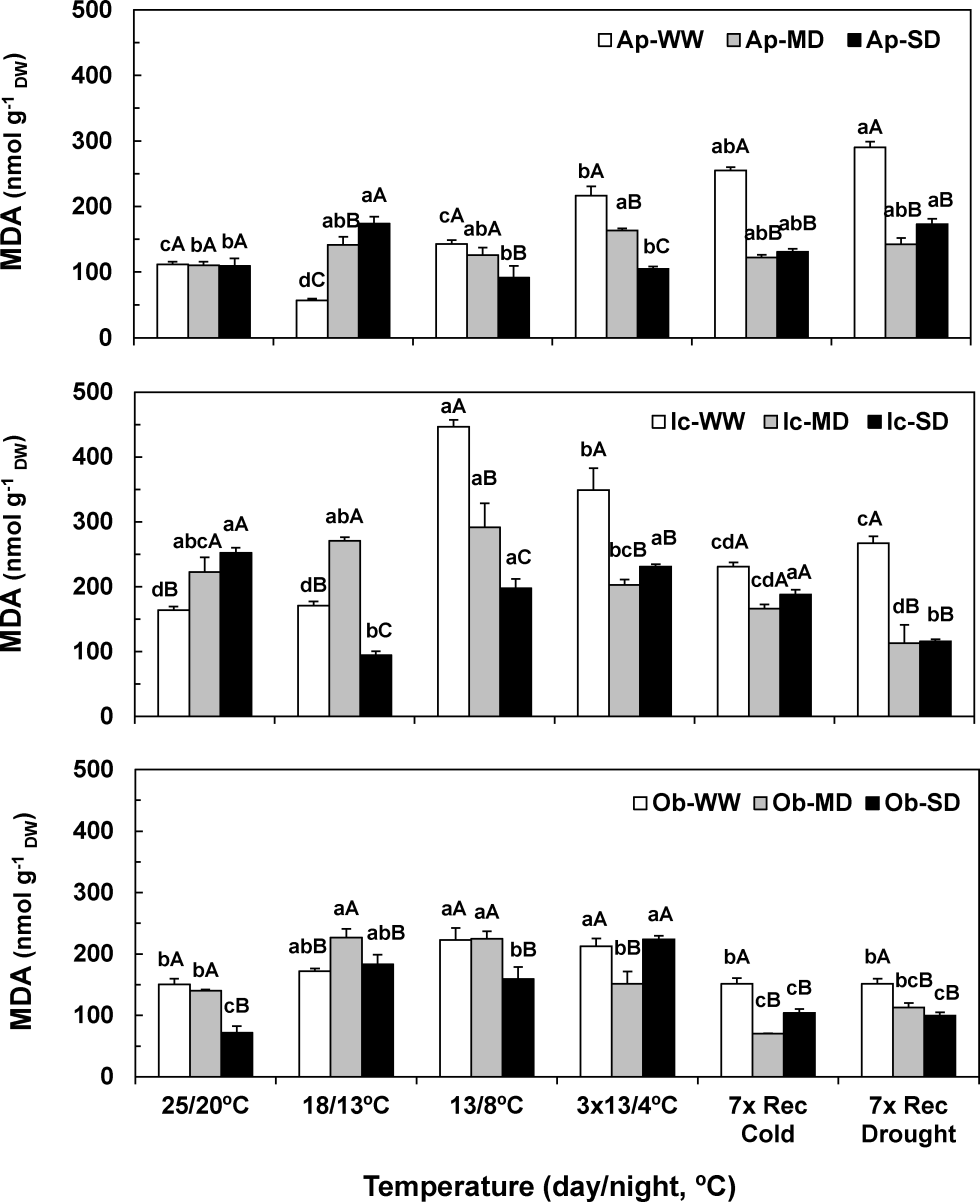
Membrane lipoperoxidation status. Changes in leaf malondialdehyde (MDA) content (nmol MDA g^-1^ dw) along the entire experiment for Apoatã (Ap), Icatu (Ic), and Obatã (Ob) genotypes, under well-watered (WW), mild drought (MD) and severe drought (SD) conditions, and submitted to temperature control conditions (25/20 ^o^C), during the gradual temperature decrease (18/13 ^o^C), at the end of the acclimation period (13/8 ^o^C), after 3 chilling cycles (3x13/4 ^o^C), after 7 days under rewarming conditions (7x Rec Cold), and after a further 7 days period under rewatering conditions (7x Rec Drought). For each parameter, the mean values ± SE (n = 4–5) followed by different letters express significant differences between temperature treatments for the same water availability level (a, b, c, d), or between water treatments for each temperature treatment (A, B, C), always separately for each genotype.

Under control temperature, drought promoted different changes in malondialdehyde (MDA) content among genotypes. While these values did not change in SD plants of Apoatã, they were increased in Icatu (54%), and reduced in Obatã (52%) plants (Fig 3). On the other hand, cold imposition alone (WW plants) increased MDA contents at 13/8°C and after chilling in all genotypes, but stronger in Icatu. However, along the recovery period MDA values tended to decline only in the *C*. *arabica* genotypes.

Again, the exposure to both stresses induced a different response than that of single stresses. An interaction was observed from 13/8°C onwards for the droughted plants of all genotypes, showing lower MDA contents than their respective WW plants. As an example, the largest MDA increase and maximal value was observed in Icatu-WW plants at 13/8°C. This content represented an increase of 173% in relation to the control temperature, and of 126% when compared to their SD plants (which showed a value close to its control). Notably, by the end of the experiment, MD and SD plants from all genotypes showed significantly lower MDA contents than their WW counterparts, and even lower contents than at the beginning of the experiment for Icatu and Obatã.

### Antioxidative enzymes

**Fig 4:**
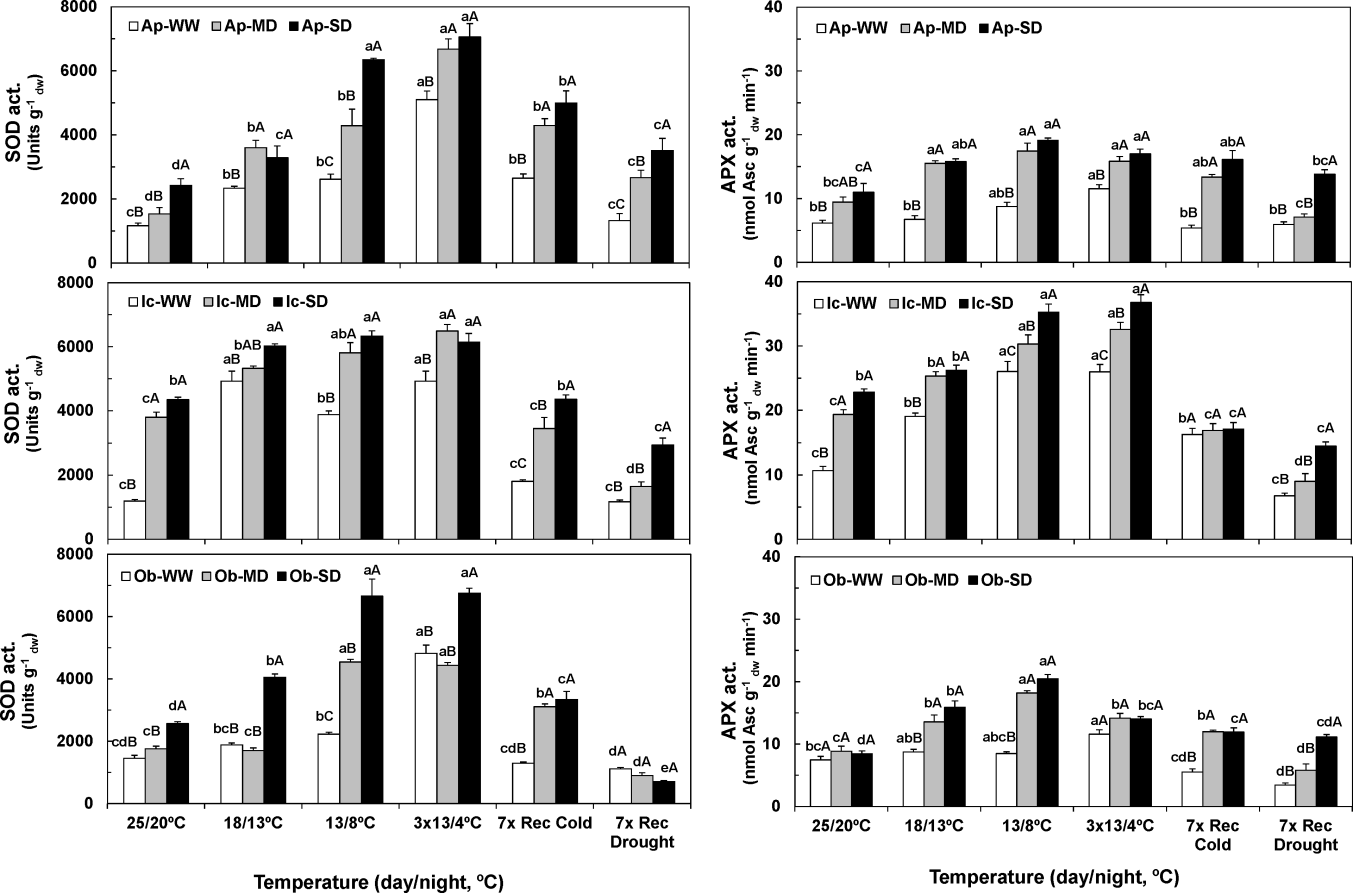
Changes in chloroplastic maximal activities of Cu,Zn-superoxide dismutase and ascorbate peroxidase. Values for the antioxidant enzymes Cu,Zn-superoxide dismutase (Cu,Zn-SOD) (left), and ascorbate peroxidase (APX) (right), along the entire experiment for Apoatã (Ap), Icatu (Ic), and Obatã (Ob) genotypes, under well-watered (WW), mild drought (MD) and severe drought (SD) conditions, and submitted to temperature control conditions (25/20 ^o^C), during the gradual temperature decrease (18/13 ^o^C), at the end of the acclimation period (13/8 ^o^C), after 3 chilling cycles (3x13/4 ^o^C), after 7 days under rewarming conditions (7x Rec Cold), and after a further 7 days period under rewatering conditions (7x Rec Drought). For each enzyme, the mean activity values ± SE (n = 4) followed by different letters express significant differences between temperature treatments for the same water availability level (a, b, c, d, e), or between water treatments for each temperature treatment (A, B, C), always separately for each genotype.

**Fig 5:**
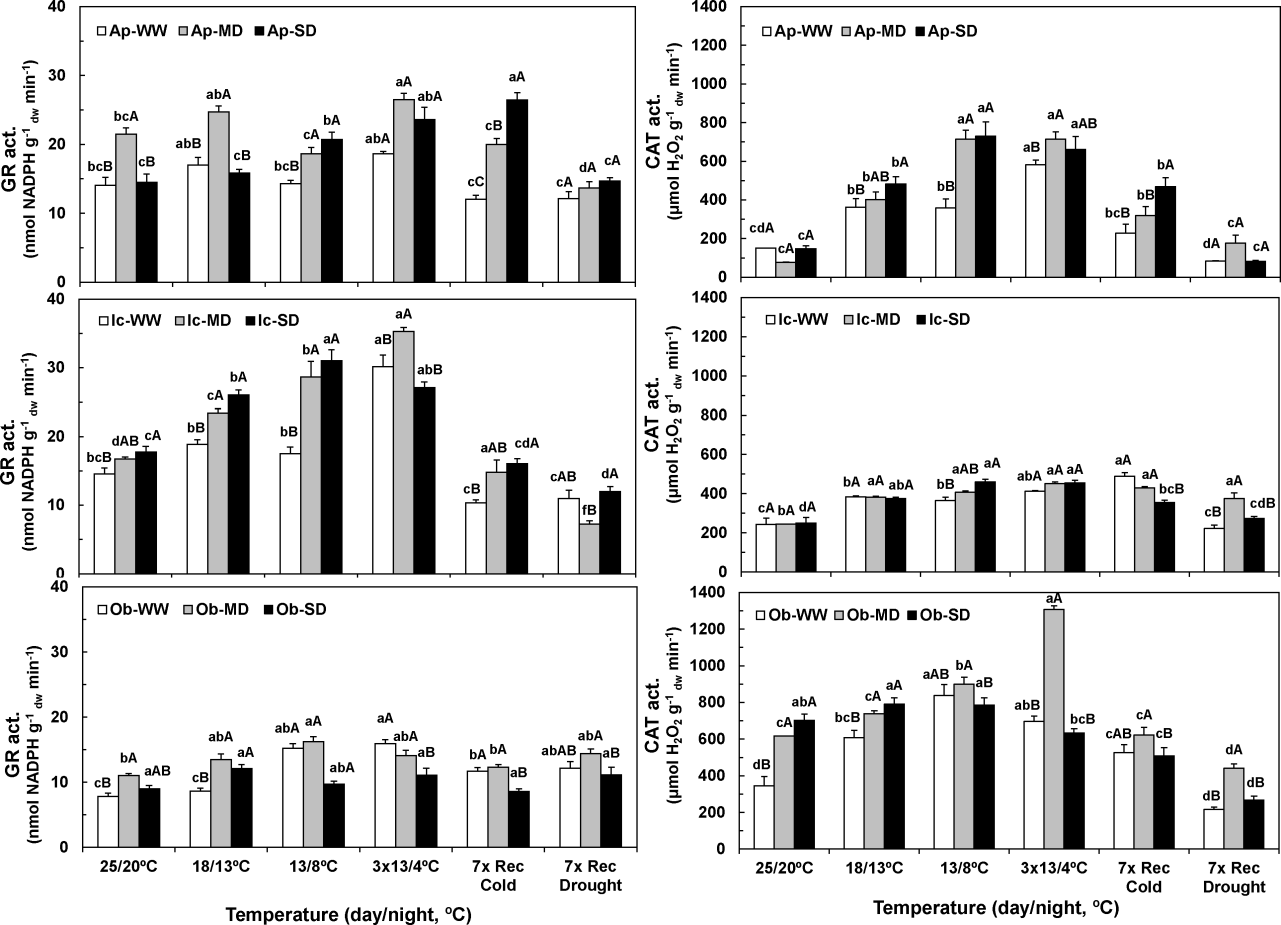
Changes in maximal activities of the chloroplastic glutathione reductase and cellular catalase. Values for antioxidant enzyme glutathione reductase (GR) (left), as well as for cellular catalase (CAT) (right), along the entire experiment for Apoatã (Ap), Icatu (Ic), and Obatã (Ob) genotypes, under well-watered (WW), mild drought (MD) and severe drought (SD) conditions, and submitted to temperature control conditions (25/20 ^o^C), during the gradual temperature decrease (18/13 ^o^C), at the end of the acclimation period (13/8 ^o^C), after 3 chilling cycles (3x13/4 ^o^C), after 7 days under rewarming conditions (7x Rec Cold), and after a further 7 days period under rewatering conditions (7x Rec Drought). For each enzyme, the mean activity values ± SE (n = 4) followed by different letters express significant differences between temperature treatments for the same water availability level (a, b, c, d, e, f), or between water treatments for each temperature treatment (A, B, C), always separately for each genotype.

The maximal cellular activities of the three enzymes contributing to remove ROS, superoxide dismutase (SOD), ascorbate peroxidase (APX) and glutathione reductase (GR), complemented to that of catalase (CAT), were greatly enhanced by both drought and cold conditions, although with some differences across genotypes (Figs 4 and 5).

Regarding each enzyme, SOD activity was incremented in all plants by drought *per se* (MD and SD plants at 25/20°C), particularly in Icatu (Fig 4), with SD plants showing the highest increases of 77%, 108% and 263% in Obatã, Apoatã and Icatu, respectively. Cold exposure alone (WW plants along the experiment) also promoted SOD activity in all genotypes, especially after chilling exposure, but Icatu showed a much stronger activity rise from 18/13°C onwards when compared with Apoatã and Obatã. However, upon both stressful conditions, an even stronger SOD activity increase was observed. Maximal values were reached after chilling, with increases of 504% (SD), 443% (MD) and 366% (SD) in Apoatã, Icatu and Obatã, respectively. Upon rewarming (7x Rec Cold) SOD activity decreased, but drought promoting effect remained. After rewatering (7x Rec Drought) SOD activity further decreased, although the previous droughted plants of Apoatã and Icatu kept increased activities, especially in SD plants that double WW values.

The effect of each individual and combined stresses in APX was also clear (Fig 4), although to a somewhat lower extent than in SOD. Icatu plants showed the highest absolute values, and the greatest increases when exposed only to drought (MD-81%; SD-114%, at 25/20°C), to cold (WW-143%, at 13/8°C and after chilling), or simultaneously to both stresses (SD-244%, after chilling). Maximal activities were found at 13/8°C and/or after chilling in the 3 genotypes, in all water conditions, with an interaction between cold and drought which further increased APX activity when compared to the impact of single stress exposure. Along the cold and water recovery periods APX activity decreased, but by the end of the experiment all ex-drought-stressed plants kept values above WW ones.

The GR activity was also responsive to the single and combined exposure to drought and cold (Fig 5). Icatu showed the greater values and increases promoted by the combination of both stresses (MD-142%, after chilling). However, it seems noteworthy that this enzyme showed most of their highest values under MD conditions, particularly at the most intense cold stress point (3x 13/4°C), and that in Icatu and Obatã the SD values were lower than those from WW plants. After cold removal GR activity clearly approached the 25/20°C initial values in all genotypes (except in SD plants of Apoatã), whereas after rewatering all plants showed similar or inferior values than their respective controls.

Drought promoted CAT activity only in Obatã MD (79%) and SD (104%) plants (Fig 5). Cold alone also boosted CAT activity in WW plants of all genotypes, strongly in Apoatã and Obatã. Additionally, a synergistic effect of cold and drought occurred, mostly for Apoatã (MD and SD) at 13/8°C, in Obatã (MD) after chilling, and in Icatu (SD) at 13/8°C.

### Non-enzymatic antioxidant molecules

Apoatã plants showed the highest constitutive *α*-tocopherol (TOC) throughout the experiment (Table 4). However, it was the only genotype with TOC decreases (16.5%) under severe drought at 25/20°C, and without increases under cold, although with a rise in the recovery period in previously droughted plants. On the other hand, TOC contents were moderately increased by drought, and strongly enhanced by cold from 13/8°C onwards, and by the combined stress exposure (after chilling) in *C*. *arabica* genotypes. After the recovery periods all water treatments (except WW in Apoatã) showed higher TOC values than their respective control (WW, 25/20°C).

**Table 4:** Variation of the leaf contents of *α*-tocopherol and ascorbate. Values of *α*-tocopherol, and ascorbate (mg g^-1^ dw) along the entire experiment for Apoatã, Icatu, and Obatã genotypes, under well-watered (WW), mild drought (MD) and severe drought (SD) conditions, and submitted to temperature control conditions (25/20 ^o^C), during the gradual temperature decrease (18/13 ^o^C), at the end of the acclimation period (13/8 ^o^C), after 3 chilling cycles (3x13/4 ^o^C), after 7 days under rewarming conditions (7x Rec Cold), and after a further 7 days period under rewatering conditions (7x Rec Drought).

Genotype	Treatment	Temperature (day/night)																							
		25/20°C				18/13°C				13/8°C				3x13/4°C				7x Rec Cold				7x Rec Drought			
		**α-Tocopherol (mg g**^**-1**^ **dw)**																							
	**WW**	2.04	±	0.05	aB	1.74	±	0.04	bcA	1.99	±	0.04	abA	1.64	±	0.04	cB	1.85	±	0.03	abcB	2.02	±	0.03	aC
**Apoatã**	**MD**	2.49	±	0.06	bA	1.96	±	0.04	cA	2.01	±	0.03	cA	1.53	±	0.03	dB	1.82	±	0.06	cB	3.15	±	0.07	aB
	**SD**	1.70	±	0.05	dC	1.18	±	0.10	eB	1.44	±	0.08	deB	2.11	±	0.04	cA	3.53	±	0.16	bA	4.19	±	0.09	aA
	**WW**	0.69	±	0.01	bcB	0.57	±	0.01	cB	1.64	±	0.05	aA	0.80	±	0.02	bcC	1.55	±	0.10	aA	1.00	±	0.04	bA
**Icatu**	**MD**	1.20	±	0.03	bA	0.84	±	0.01	cA	1.33	±	0.02	bB	1.75	±	0.13	aA	1.28	±	0.04	bB	1.07	±	0.03	bcA
	**SD**	0.89	±	0.02	cdB	0.79	±	0.02	dAB	1.08	±	0.04	abcdC	1.32	±	0.07	aB	1.20	±	0.04	abcB	0.99	±	0.02	bcdA
	**WW**	0.54	±	0.02	dB	0.62	±	0.02	cdA	0.90	±	0.01	bA	0.86	±	0.03	bcCB	1.40	±	0.04	aB	1.49	±	0.03	aA
**Obatã**	**MD**	0.78	±	0.02	dA	0.78	±	0.02	dA	0.83	±	0.03	cdA	1.08	±	0.02	bcAB	1.16	±	0.04	abC	1.37	±	0.05	aA
	**SD**	0.84	±	0.04	deA	0.66	±	0.03	eA	1.00	±	0.05	dA	1.29	±	0.04	cA	1.99	±	0.04	aA	1.55	±	0.08	bA
		**Ascorbate (mg g**^**-1**^ **dw)**																							
	**WW**	0.83	±	0.11	cA	2.14	±	0.21	abAB	2.52	±	0.18	aA	1.84	±	0.17	bA	2.07	±	0.08	abAB	1.13	±	0.13	cB
**Apoatã**	**MD**	0.45	±	0.23	cA	2.45	±	0.20	aA	2.83	±	0.15	aA	1.71	±	0.21	bA	1.71	±	0.21	bB	2.74	±	0.23	aA
	**SD**	0.85	±	0.09	dA	1.77	±	0.16	bcB	1.39	±	0.11	cB	1.94	±	0.20	abA	2.19	±	0.19	abA	2.45	±	0.14	aA
	**WW**	0.88	±	0.04	cAB	1.48	±	0.08	bA	1.88	±	0.34	abA	2.45	±	0.14	aA	2.17	±	0.22	aA	2.21	±	0.21	aA
**Icatu**	**MD**	1.14	±	0.15	bA	1.29	±	0.10	abA	1.83	±	0.08	aA	1.47	±	0.07	abB	1.13	±	0.05	bB	1.22	±	0.13	abB
	**SD**	0.49	±	0.08	bB	1.03	±	0.20	abA	1.63	±	0.14	aA	1.85	±	0.12	aB	0.93	±	0.30	bB	0.71	±	0.05	bC
	**WW**	1.42	±	0.39	cdAB	1.13	±	0.20	dB	1.83	±	0.24	abcA	1.71	±	0.07	bcdA	1.88	±	0.53	abcA	2.52	±	0.14	aA
**Obatã**	**MD**	1.82	±	0.29	abA	2.10	±	0.22	aA	1.09	±	0.08	cdB	0.52	±	0.08	dB	1.36	±	0.22	bcA	1.84	±	0.35	abB
	**SD**	1.04	±	0.07	bB	0.88	±	0.11	bB	1.06	±	0.16	bB	1.04	±	0.30	bB	1.84	±	0.46	aA	1.41	±	0.44	abB

For each parameter, the mean values ± SE (n = 6–8) followed by different letters express significant differences between temperature treatments for the same water availability level (a, b, c, d, e), or between water treatments for each temperature treatment (A, B, C), always separately for each genotype.

As regards ascorbate (ASC), Obatã presented the highest constitutive values, and together with Icatu, showed a 29% increase related to drought (MD at 25/20°C) (Table 4). Cold promoted ASC synthesis in all genotypes, particularly in Apoatã and Icatu at 13/8°C and after 4°C exposure. For all genotypes, no stress interaction was observed since MD and SD plants presented similar or lower values (usually Obatã) than WW plants upon 13/8°C or after chilling. After both stresses removal, increased ASC contents were maintained in Apoatã (all water treatments), Icatu and Obatã (WW plants), when compared to the initial control plants (WW, 25/20°C).

Drought *per se* promoted significant zeaxanthin (ZEA) synthesis in MD plants (but not in SD) of Apoatã and Icatu (142% and 43%, respectively), whereas cold promoted a greater ZEA accumulation, with the highest increases of 270 and 173% in WW plants at 13/8°C, in the same genotype order (Table 5). The combined stress exposure resulted in even higher ZEA contents in some cases, namely in Obatã (MD and SD at 18/13°C and after chilling), and Apoatã (MD and SD, from 18/13°C until after chilling exposure). ZEA increases resulted from the transformation of existing VIOL and ANT, but also from a reinforcement of the xanthophyll cycle pool content (VIOL+ANT+ZEA) promoted mostly by cold and by stress interaction in Apoatã and Icatu. In accordance with ZEA rise, the de-epoxidation state (DEPS) increased due to the single exposure to drought (except Obatã) and cold. The exposure to both stresses caused an even greater DEPS rise, mostly at 18/13°C. However, only a few differences between water treatments were observed at 13/8°C and after chilling because DEPS value was almost saturated (close or above 0.9) in all of them. Along the cold and drought recovery periods ZEA, V+A+Z and DEPS approached control values, but by the end of the experiment Apoatã and Icatu tended to somewhat higher values of ZEA and DEPS, especially in MD and/or SD plants.

**Table 5:** Variation of the leaf contents regarding the xanthophylls cycle components. Values of zeaxanthin and the sum of the xanthophylls violaxanthin, antheraxanthin and zeaxanthin (V+A+Z) (mg g^-1^ dw), as well as the xanthophylls de-epoxidation state (DEPS) along the entire experiment for Apoatã, Icatu, and Obatã genotypes, under well-watered (WW), mild drought (MD) and severe drought (SD) conditions, and submitted to temperature control conditions (25/20 ^o^C), during the gradual temperature decrease (18/13 ^o^C), at the end of the acclimation period (13/8 ^o^C), after 3 chilling cycles (3x13/4 ^o^C), after 7 days under rewarming conditions (7x Rec Cold), and after a further 7 days period under rewatering conditions (7x Rec Drought).

Genotype	Treatment	Temperature (day/night)																							
		25/20°C				18/13°C				13/8°C				3x13/4°C				7x Rec Cold				7x Rec Drought			
		**Zeaxanthin (mg g**^**-1**^ **dw) (diurnal values)**																							
	**WW**	0.069	±	0.008	dB	0.181	±	0.015	bB	0.256	±	0.016	aB	0.167	±	0.015	bcB	0.085	±	0.002	dA	0.117	±	0.010	cdA
**Apoatã**	**MD**	0.167	±	0.007	bcA	0.291	±	0.029	aA	0.334	±	0.019	aA	0.205	±	0.024	bAB	0.069	±	0.015	dA	0.129	±	0.008	cdA
	**SD**	0.077	±	0.011	cB	0.221	±	0.022	bB	0.308	±	0.026	aAB	0.235	±	0.009	bA	0.122	±	0.003	cA	0.136	±	0.007	cA
	**WW**	0.146	±	0.018	bcB	0.178	±	0.008	bA	0.400	±	0.027	aA	0.336	±	0.025	aAB	0.153	±	0.009	bcA	0.100	±	0.012	cB
**Icatu**	**MD**	0.210	±	0.039	bcA	0.186	±	0.007	cA	0.343	±	0.014	aAB	0.267	±	0.012	bB	0.153	±	0.009	cA	0.194	±	0.010	cA
	**SD**	0.153	±	0.007	bB	0.199	±	0.012	bA	0.326	±	0.018	aB	0.368	±	0.014	aA	0.155	±	0.007	bA	0.177	±	0.016	bA
	**WW**	0.187	±	0.010	bA	0.211	±	0.028	abC	0.256	±	0.009	aA	0.218	±	0.016	abB	0.120	±	0.012	cB	0.186	±	0.009	bA
**Obatã**	**MD**	0.185	±	0.007	eA	0.342	±	0.016	abA	0.283	±	0.014	bcA	0.356	±	0.016	aA	0.271	±	0.012	cdA	0.215	±	0.018	deA
	**SD**	0.209	±	0.018	dA	0.282	±	0.015	bcB	0.304	±	0.016	abA	0.361	±	0.014	aA	0.234	±	0.009	cdA	0.211	±	0.019	dA
		**Violaxanthin + Antheraxanthin + Zeaxanthin (mg g**^**-1**^ **dw) (diurnal values)**																							
	**WW**	0.222	±	0.008	bcA	0.284	±	0.015	abB	0.319	±	0.019	aB	0.213	±	0.014	cB	0.210	±	0.004	cB	0.226	±	0.005	bcA
**Apoatã**	**MD**	0.273	±	0.017	cdA	0.344	±	0.030	abA	0.403	±	0.010	aA	0.211	±	0.024	dB	0.286	±	0.021	bcA	0.238	±	0.007	cdA
	**SD**	0.222	±	0.009	bcA	0.299	±	0.022	aAB	0.350	±	0.027	aAB	0.283	±	0.014	abA	0.212	±	0.015	cB	0.193	±	0.008	cA
	**WW**	0.314	±	0.012	bcA	0.274	±	0.012	cdA	0.436	±	0.021	aA	0.358	±	0.024	bA	0.303	±	0.019	bcdB	0.233	±	0.007	dB
**Icatu**	**MD**	0.343	±	0.027	abA	0.237	±	0.009	cA	0.409	±	0.013	aAB	0.397	±	0.015	aA	0.380	±	0.017	abA	0.320	±	0.008	bA
	**SD**	0.304	±	0.027	bcA	0.267	±	0.011	cA	0.361	±	0.021	abB	0.403	±	0.012	aA	0.329	±	0.014	bcAB	0.322	±	0.031	bcA
	**WW**	0.323	±	0.010	bcB	0.264	±	0.022	cB	0.287	±	0.010	bcB	0.267	±	0.013	cB	0.408	±	0.026	aA	0.342	±	0.013	bA
**Obatã**	**MD**	0.450	±	0.009	aA	0.394	±	0.016	abcA	0.338	±	0.015	cAB	0.411	±	0.021	abA	0.438	±	0.009	aA	0.347	±	0.020	bcA
	**SD**	0.463	±	0.024	aA	0.391	±	0.008	bcA	0.370	±	0.012	bcA	0.429	±	0.022	abA	0.392	±	0.011	bcA	0.329	±	0.011	cA
		**DEPS**																							
	**Ctr**	0.427	±	0.024	dB	0.698	±	0.021	bB	0.838	±	0.021	aB	0.843	±	0.028	aB	0.532	±	0.008	cB	0.575	±	0.035	cC
**Apoatã**	**MD**	0.645	±	0.022	cA	0.837	±	0.019	bA	0.869	±	0.028	abAB	0.941	±	0.012	aA	0.378	±	0.030	dC	0.665	±	0.019	cB
	**SD**	0.511	±	0.025	eB	0.814	±	0.035	bcA	0.911	±	0.013	aA	0.884	±	0.013	abAB	0.699	±	0.026	dA	0.781	±	0.009	cA
	**Ctr**	0.495	±	0.039	cB	0.770	±	0.019	bB	0.918	±	0.010	aA	0.950	±	0.005	aA	0.651	±	0.044	bA	0.477	±	0.036	cB
**Icatu**	**MD**	0.632	±	0.049	bcA	0.895	±	0.015	aA	0.891	±	0.013	aA	0.943	±	0.011	aA	0.539	±	0.016	cB	0.705	±	0.017	bA
	**SD**	0.652	±	0.019	bA	0.859	±	0.018	aAB	0.942	±	0.004	aA	0.956	±	0.004	aA	0.618	±	0.016	bAB	0.704	±	0.025	bA
	**Ctr**	0.668	±	0.012	cA	0.822	±	0.041	bB	0.916	±	0.012	aA	0.875	±	0.011	abA	0.562	±	0.046	dB	0.679	±	0.030	cA
**Obatã**	**MD**	0.516	±	0.016	cB	0.908	±	0.008	aA	0.884	±	0.009	aA	0.906	±	0.008	aA	0.708	±	0.022	bA	0.716	±	0.023	bA
	**SD**	0.551	±	0.020	cB	0.839	±	0.013	aAB	0.892	±	0.014	aA	0.885	±	0.014	aA	0.702	±	0.018	bA	0.710	±	0.030	bA

For each parameter, the mean values ± SE (n = 6–8) followed by different letters express significant differences between temperature treatments for the same water availability level (a, b, c, d, e), or between water treatments for each temperature treatment (A, B, C), always separately for each genotype.

Total phenol content (TPC) was only moderately increased by mild water deficit in Icatu (18%) and Obatã (22%), under control temperature (Table 6). Some significant increases were promoted by cold in WW plants, with maximal increases of 20% in Apoatã (13/8°C), 22% in Icatu (after chilling), and close or above 50% in Obatã at 18/13°C and onwards. Stresses interaction resulted in additional increases in Icatu MD plants throughout the entire experiment, and in Apoatã SD plants from chilling exposure onwards. By the end of the experiment only Obatã (all water conditions) and Icatu (MD) kept values above control.

**Table 6:** Changes in the leaf contents of total phenols and 5-caffeoylquinic acid. Values of total phenols (mg GAE g^-1^ dw), and 5-caffeoylquinic acid (5-CQA) along the entire experiment for Apoatã, Icatu, and Obatã genotypes, under well-watered (WW), mild drought (MD) and severe drought (SD) conditions, and submitted to temperature control conditions (25/20 ^o^C), during the gradual temperature decrease (18/13 ^o^C), at the end of the acclimation period (13/8 ^o^C), after 3 chilling cycles (3x13/4 ^o^C), after 7 days under rewarming conditions (7x Rec Cold), and after a further 7 days period under rewatering conditions (7x Rec Drought).

Genotype	Treatment	Temperature (day/night)																							
		25/20°C				18/13°C				13/8°C				3x13/4°C				7x Rec Cold				7x Rec Drought			
		**Total Phenols (mg GAE g**^**-1**^ **dw)**																							
	**WW**	139.1	±	1.3	cA	153.3	±	1.5	bC	166.9	±	1.1	aA	148.1	±	1.6	bB	162.1	±	2.8	aB	137.8	±	1.7	cB
**Apoatã**	**MD**	107.1	±	1.6	cB	170.9	±	2.1	aB	147.0	±	1.2	bC	93.9	±	2.0	dC	52.0	±	0.3	eC	99.2	±	1.2	dC
	**SD**	134.9	±	1.3	cA	185.5	±	0.9	aA	159.8	±	1.7	bB	161.4	±	2.2	bA	189.0	±	1.5	aA	154.4	±	1.1	bA
	**WW**	98.4	±	0.6	bB	68.0	±	0.7	cB	96.3	±	5.0	bB	120.0	±	6.8	aB	88.6	±	5.8	bB	90.0	±	2.9	bB
**Icatu**	**MD**	116.1	±	1.1	cA	106.7	±	0.4	cA	169.9	±	1.5	aA	136.0	±	0.3	bA	112.4	±	1.7	cA	115.8	±	1.6	cA
	**SD**	75.0	±	0.5	bC	42.5	±	0.2	cC	101.9	±	0.9	aB	110.3	±	1.4	aB	53.7	±	0.5	cB	83.1	±	0.8	bB
	**WW**	63.4	±	0.9	eB	128.3	±	2.1	aA	93.8	±	0.5	dB	102.7	±	0.9	cA	114.8	±	1.1	bC	123.6	±	1.0	aA
**Obatã**	**MD**	77.1	±	1.0	eA	65.5	±	0.6	fC	93.3	±	0.9	cB	100.7	±	1.1	bA	163.4	±	0.6	aA	85.2	±	0.4	dB
	**SD**	60.5	±	0.7	eB	89.1	±	1.0	cB	114.4	±	1.3	bA	86.8	±	0.8	cdB	122.1	±	0.8	aB	82.6	±	1.0	dB
		**5-CQA (mg g**^**-1**^ **dw)**																							
	**WW**	18.5	±	0.6	bA	22.2	±	0.5	abB	24.4	±	0.6	aAB	24.3	±	0.5	aA	24.7	±	0.6	aB	13.3	±	0.3	cB
**Apoatã**	**MD**	15.6	±	0.3	bB	14.1	±	0.4	bcC	22.3	±	0.6	aB	9.5	±	0.4	dC	2.0	±	0.1	eC	10.8	±	0.3	cdB
	**SD**	13.8	±	0.5	cB	29.1	±	1.0	abA	25.7	±	0.6	aA	18.4	±	0.7	bB	27.9	±	0.6	aA	19.9	±	0.7	bA
	**WW**	25.0	±	1.1	bA	17.9	±	0.4	cB	26.8	±	0.3	bB	39.7	±	1.1	aA	23.6	±	0.3	bA	25.5	±	0.8	bA
**Icatu**	**MD**	23.0	±	0.5	bA	22.1	±	0.3	bA	31.8	±	0.5	aA	28.3	±	0.4	aB	22.8	±	0.4	bA	20.5	±	0.5	bB
	**SD**	14.0	±	0.2	bB	6.2	±	0.2	cC	20.6	±	0.6	aC	21.1	±	0.2	aC	7.8	±	0.2	cB	15.2	±	0.4	bC
	**WW**	16.5	±	0.4	cA	23.6	±	0.2	bA	23.1	±	0.4	bB	23.2	±	0.5	bA	31.0	±	0.6	aA	25.5	±	0.5	bA
**Obatã**	**MD**	12.1	±	0.5	dB	17.5	±	0.4	cB	19.4	±	0.5	bC	25.2	±	0.8	aA	21.7	±	0.3	abB	18.5	±	0.3	bcB
	**SD**	13.3	±	0.3	dB	22.0	±	0.3	bA	28.5	±	0.7	aA	17.8	±	0.3	cB	20.0	±	0.3	bcB	16.5	±	0.4	cB

For each parameter, the mean values ± SE (n = 5) followed by different letters express significant differences between temperature treatments for the same water availability level (a, b, c, d, e, f), or between water treatments for each temperature treatment (A, B, C), always separately for each genotype.

In all genotypes 5-CQA content decreased under drought (significantly in SD plants), whereas increased with cold exposure at 13/4°C and after chilling (Table 6). Stress interaction was observed only in few cases (Icatu MD plants at 18/13°C and 13/8°C; Apoatã SD at 18/13°C; Obatã SD at 13/8°C). Still, in all genotypes after chilling, 5-CQA usually decreased in droughted plants when compared to the values under cold alone.

### Expression of genes with a potential role in drought and cold acclimation

The transcriptional patterns of genes encoding for key enzymes for ROS scavenging was studied, regarding APX for H_2_O_2_ removal [(*APXc* (cytosolic), *APXm* (membrane-bound), and *APXt+s* (stromatic)], for energy dissipation in the photosystems through ZEA synthesis by violaxanthin de-epoxidase, VDE (*VDE2*), and one class III peroxidase (*PX4*) (Table 1).

Drought (at 25/20°C) promoted the expression of the three studied APX genes, in Apoatã and Icatu (except *APXm*), as compared to their respective values of WW plants, but only in Icatu significant increases were observed for *APXc* and *APXt+s* (Table 7).

**Table 7:** Changes in gene transcription. Real-time-qPCR expression values (n fold) relative to the expression value observed under control conditions of temperature (25/20°C) and water availability (WW), within each genotype. The values are for the entire experiment from leaves of Apoatã, Icatu, and Obatã genotypes, under well-watered (WW), mild drought (MD) and severe drought (SD) conditions, and submitted to temperature control conditions (25/20 ^o^C), at the end of the acclimation period (13/8 ^o^C), after 3 chilling cycles (3x13/4 ^o^C), after 7 days under rewarming conditions (7x Rec Cold), and after a further 7 days period under rewatering conditions (7x Rec Drought). It were studied genes of the enzymes ascorbate peroxidases from cytosolic ascorbate peroxidase (*APXc*), membrane-bound ascorbate peroxidase (*APXm*), and stromatic ascorbate peroxidase (*APXt+s*), peroxidase (*PX4*), and violaxanthin de-epoxidase (*VDE2*).

			Gene Expression Relative to Control Conditions				
Genotype	Temperature	Water	*APXc*	*APXm*	*APXt+s*	*PX4*	*VDE2*
**Apoatã**	**25/20°C**	**WW**	1.00	1.00	1.00	1.00	1.00
		**MD**	2.96	2.76	3.03	3.13	2.84
		**SD**	2.85	2.20	2.13	5.73*	4.07
	**13/8°C**	**WW**	1.37	1.40	1.54	2.87	0.86
		**MD**	3.16	2.62	4.45*	6.34*	1.69
		**SD**	0.67	0.57	0.69	3.75	3.63
	**3 x 13/4°C**	**WW**	1.53	1.05	1.54	0.12*	0.92
		**MD**	2.02	2.55	2.24	1.31	0.30
		**SD**	1.42	1.02	1.89	1.24	1.46
	**7x Rec Cold**	**WW**	3.90	1.78	3.80	6.95*	1.52
		**MD**	7.02*	3.01	3.93	2.43	2.61
		**SD**	2.29	1.76	2.28	4.39	1.80
	**7x Rec Drought**	**WW**	2.15	1.59	1.80	6.98*	1.13
		**MD**	3.53	2.61	1.82	8.16*	1.97
		**SD**	3.54	1.79	1.82	9.42*	2.64
**Icatu**	**25/20°C**	**WW**	1.00	1.00	1.00	1.00	1.00
		**MD**	6.43*	1.39	4.18*	15.24*	0.25
		**SD**	6.63*	0.76	1.75	0.67	0.24
	**13/8°C**	**WW**	1.86	0.32	1.16	2.11	0.15
		**MD**	2.20	0.31	1.53	6.56*	0.07*
		**SD**	10.28*	1.91	3.22	10.54*	0.69
	**3 x 13/4°C**	**WW**	1.05	0.21	0.74	2.30	0.07*
		**MD**	2.88	0.41	1.12	3.55	0.09*
		**SD**	3.98*	0.72	2.27	8.50*	0.14
	**7x Rec Cold**	**WW**	1.20	0.30	0.86	4.60	0.15
		**MD**	1.84	0.31	0.83	2.67	0.11*
		**SD**	5.51*	0.69	2.60	4.86*	0.45
	**7x Rec Drought**	**WW**	0.92	0.15*	0.47	4.27	0.23
		**MD**	3.17	0.20	0.77	7.68*	0.29
		**SD**	7.54*	0.31	1.66	9.93*	0.10*
**Obatã**	**25/20°C**	**WW**	1.00	1.00	1.00	1.00	1.00
		**MD**	1.45	1.34	2.29	0.71	1.61
		**SD**	0.92	1.03	0.29	0.59	1.19
	**13/8°C**	**WW**	0.88	0.91	1.42	1.70	1.05
		**MD**	1.56	0.95	2.15	0.50	0.54
		**SD**	1.03	1.60	0.29	0.65	0.52
	**3 x 13/4°C**	**WW**	0.64	0.72	1.20	0.60	0.46
		**MD**	1.21	1.00	1.97	1.64	0.74
		**SD**	2.15	1.12	0.40	0.59	0.57
	**7x Rec Cold**	**WW**	1.00	0.68	1.23	0.44	0.46
		**MD**	0.96	0.51	1.08	0.22	0.61
		**SD**	0.93	0.71	0.26	0.27	0.93
	**7x Rec Drought**	**WW**	0.96	0.51	1.11	1.02	0.36
		**MD**	1.65	0.93	1.22	1.16	0.75
		**SD**	1.79	0.98	0.53	1.42	1.07

Original expression values for each gene resulted from the mean ± SE (n = 6–9), from 3 independent biological assays.

* indicate the presence of statistical significance.

Cold alone did not implicate significant changes in the transcriptional activity of these APX genes, but stress interaction tended to promote the upregulation of the three genes in Apoatã, especially in MD plants, what was prolonged along the recovery periods. In Icatu, this interaction promoted the highest expression increases of *APXc* until chilling exposure in droughted plants (particularly SD), being as well the only genotype to maintain significant increased upregulation until the end of the experiment in at least one of the APX genes (*APXc*). This genotype also presented a consistent tendency to higher expression of *APXt+s* in SD plants. Obatã plants showed the lowest up regulation of APX genes, without significant expression changes but with their higher values observed at 13/8°C or after chilling in MD or SD plants for *APXc* and *APXt+s*.

The class III peroxidase gene (*PX4*) was significantly upregulated with drought in Apoatã (SD) and Icatu (MD) plants. Although without significant expression increases, cold alone consistently promoted some transcript accumulation in these genotypes along the entire experiment (except in Apoatã after chilling). The stress interaction further enhanced transcriptional activity in most cases, with SD plants often showing higher values, particularly in Icatu.

*VDE2* showed a different transcript accumulation pattern between genotypes. Drought alone promoted some accumulation of transcripts in Apoatã, which was maintained under both stresses imposition (*e*.*g*., SD plants at 13/8°C). As regards the *C*. *arabica* genotypes, both cold and/or drought consistently reduced *VDE2* transcripts, usually until the end of the experiment, significantly in Icatu for several cases.

Although with some expression fluctuations, Obatã showed a different response pattern, without significant expression changes to any of the applied stress conditions for the studied gene transcripts.

## Discussion

### Drought and cold impacts on leaf water status

The slow imposition of water deficits and temperature stresses allows the triggering of a range of time-dependent morphological and physiological acclimation, and even in stress-sensitive plants some acclimation is possible [74].

Leaf dehydration was a consequence of the imposed reduction of water availability at control temperature (Table 3). Additionally, it was further promoted by cold (two lowest temperatures) in MD and/or SD plants, and, especially, in WW plants, resulting in closer RWC values between water conditions in all genotypes. As drought, cold can promote cell dehydration [75,76], namely, by reducing root water uptake [77]. Accordingly, many cold acclimation responses are linked to dehydration [78,79], being similar to those observed in woody plants under drought [6,76].

Icatu might have displayed some osmotic adjustment capability, an uncommon trait among coffee genotypes under drought [30], showing the lowest Ψ_w_ values for MD and SD plants in some temperatures (*e*.*g*., 13/8°C) but similar RWC values to the other genotypes. Also, Icatu MD and SD plants recovered better, in line with earlier reports of cold tolerance [42,45]. This tolerance was also reflected in the absence of leaf senescence in MD and SD plants along cold exposure (and afterwards), when compared to WW plants (data not shown). A similar stress cross-tolerance was reported in *Camelis sinensis* (L.) O. Kuntze, where drought-induced leaf senescence was delayed by cold superimposition, due to, namely, enhanced antioxidant capacity, attenuated lipid degradation, and maintenance of the photosynthetic system [76], as found in Icatu plants (see below).

### Drought and cold impairments on photosynthesis and membranes

In coffee, photosynthesis become limited below 18°C, and both stomata and mesophyll impacts on the photosynthetic apparatus occur under chilling [42,43,45], although in a genotype dependent manner. In addition, depending on the duration and severity of stress, relevant drought impacts can be expected at stomata and mesophyll levels [30,78]. This agrees with our findings, since leaf gas exchanges were clearly disturbed by the single and combined drought and cold exposure in all genotypes (Fig 2). Each stress decreased P_n_, related to stomatal closure (data not shown), and reduction of biochemical reactions at 18°C (A_max_), confirming earlier findings for cold [42,45]. Notably, a greater limitation was imposed by cold than by drought to the photosynthetic functioning, as reflected in the much larger P_n_ and A_max_ reductions driven by cold in all genotypes, in accordance to the cold sensitivity displayed by most tropical and sub-tropical plants [50]. Nevertheless, although close patterns were observed in all genotypes, tolerance differences were clear. Apoatã was the most affected genotype from 18/13°C onwards, with aftereffects persisting in A_max_ by the end of the experiment (although with a total P_n_ recovery), in line with its cold sensitivity as compared to Icatu, related to lower membrane stability and incomplete triggering of protective mechanisms [45,49,57]. In contrast, Icatu plants maintained positive C-assimilation rates along cold exposure (even after chilling), and totally recovered P_n_ and A_max_ by the end of the experiment, showing lower sensitivity to each stress and to their interaction. Remarkably, the previous exposure to water shortage (MD and SD) mitigated the chilling impact at mesophyll level (reflected in A_max_) in *C*. *arabica* genotypes when compared to WW plants. Even Apoatã showed a positive response to stress interaction, as droughted plants showed a faster P_n_ recovery after cold stress removal than WW ones. A lower impact on photosynthesis under this combined exposure of stresses than only to drought was also reported in *Glycine max* (L.) Merril [75], showing that plants respond differently to multiple interacting stresses than to single stressors [4]. Such lower impact under the combined stress exposure was further reflected in the reduced MDA contents in MD and SD plants from 13/8°C onwards, particularly in Icatu and Apoatã (Fig 3). MDA is a secondary end product of the oxidation of polyunsaturated fatty acids by ROS, being a useful proxy of general lipid peroxidation, and of stress sensitivity [80–82]. Notably, by the end of the experiment, all previously droughted plants showed lower lipoperoxidation level than WW plants, being even below the initial constitutive levels in Icatu and Obatã. This was in line with what is found in chilling tolerant species after prolonged low temperatures exposure [15,17], and may have included qualitative changes in membrane lipids [47,48], turning them less susceptible to peroxidative attack by ROS or degradative enzymes, thus, decreasing MDA levels.

### Antioxidative enzyme defences reinforcement by drought and cold exposure

A wide number of studies linked high antioxidant enzyme activity with environmental stress tolerance, namely to drought [20,83], and cold [14,21,84], including in coffee [41,49,51]. Chloroplasts are a major cellular source of ROS [13] that must be promptly scavenged to protect thylakoids and stroma targets. The ascorbate-glutathione cycle is an important part of the chloroplast antioxidative system that includes several enzymes and non-enzyme molecules acting in an integrated manner [14,19,21]. Also, extra-chloroplastic detoxification systems involving catalase and phenolic reductants (*e*.*g*., CGA) complementary act as H_2_O_2_ scavenging pathways, since this ROS is relatively stable and capable to diffuse across membranes from their site of generation [14,17].

All genotypes usually showed a global triggering of the activities of the studied antioxidative enzymes, due to the single exposure to drought (except CAT in Apoatã and Icatu) and cold (Figs 4 and 5). This shows the complementary response of these antioxidative enzymes, and reflects a common response among *Coffea* spp. Moreover, a clear drought and cold interaction further reinforced these antioxidative enzymes activity, while reduced the MDA level in MD and SD plants of all genotypes. In some cases, the stress interaction surpassed a simple additive effect of each single stress (usually at 13/8°C), pointing to a synergistic increase (found also in the transcription of genes encoding for APX). Notably, the MD and/or SD plants of Icatu showed the highest values of SOD (with Apoatã and Obatã), APX, and GR, as well the largest increases at 13/8°C or after chilling exposure, justifying the globally lower lipoperoxidation impact along cold exposure. Such enhanced antioxidant capability in MD and/or SD plants was partly kept along the recovery periods (SOD and APX), likely conferring an advantage in face of new water deficit and/cold episodes. Interestingly, Icatu showed the lowest CAT activity increments due to cold, and the greatest APX activity response, in line with similar findings in *C*. *arabica* cv. Catuaí [49]. This contrasted with the trends observed in Apoatã and Obatã, and suggests a somewhat diverse path for H_2_O_2_ scavenging among coffee genotypes, with a stronger control in Icatu at chloroplast (APX) level.

It is known that ROS accumulation alters the redox potential that is implicated in gene induction [77,85]. Also, drought and cold tolerance responses share signaling transduction pathways and molecular connections, which crosstalk between them [77,79,86,87]. It is relevant that the expression patterns of the genes coding for enzymes related to antioxidative mechanisms only partly followed the pattern of enzyme activities, closer and stronger in Apoatã and, especially, Icatu. Contrary to enzyme activities, drought was a stronger promoter of transcriptional activity than cold in Apoatã and Icatu for these genes, considering an upregulation close to, or higher than 2 fold (Table 7). Also, Icatu WW plants showed the lowest transcript accumulation regarding the APX coding genes under cold (contrasting with the higher activity increase), whereas in Apoatã the opposite situation was observed. This can be related to the involvement of different genes, implicating that genes from the same family can be either down- or up-regulated, as in the case of *CcAPX2* and *CcAPX1*, respectively, in *C*. *canephora* under drought [88]. Moreover, under the simultaneous stress exposure a molecular crosstalk might result in the co-activation of different stress response pathways, promoting synergistic or antagonistic responses in several species [4,6,7]. In fact, the stress interaction reinforced the expression (and APX activity) of *APXc*, *APXt+s*, *PX4* in Icatu (*e*.*g*., SD plants at 13/8°C), followed by Apoatã, whereas Obatã was the less responsive genotype considering the studied genes.

### Complementary non-enzymatic antioxidant molecules

Plants have non-enzyme molecules that scavenge highly reactive molecules (of Chl and oxygen), among them TOC, ASC and ZEA, which further contribute to abiotic stress acclimation, namely to chilling and drought [14,27,89,90]. TOC is the major lipophilic antioxidant present in the thylakoid membrane lipid bilayer, and a membrane stabilizing agent [21–23], whereas ASC removes ROS together with APX, and non-enzymatically. TOC and ASC showed a strong dynamics in the coffee plants (Table 4), being somewhat responsive to moderate drought, and highly promoted by cold in all genotypes (except TOC in Apoatã). This likely reflects a greater relevance of non-enzyme antioxidants under low temperature, when enzyme reactions are repressed [10,14,23]. Notably, TOC and ASC content were commonly increased by drought (MD), and cold (13/8°C and/or after chilling) only in Icatu, which showed also the highest responsiveness in WW (ASC) and MD (TOC) at the harsh chilling conditions. This simultaneous ASC and TOC response could assume an extra importance since ASC can reduce the oxidized form of TOC, improving TOC antioxidant capabilities in non-aqueous phases [16]. High ASC levels also improve ZEA protection, since it is used by violaxanthin de-epoxidase (VDE) to form ZEA from violaxanthin [14,21], whereas TOC can be even more important when the xanthophyll cycle-dependent energy dissipation is saturated, and extra photoprotection is required [23,49]. This was the case for all genotypes, as DEPS values were close or above to 0.9 from 18/13°C until 4°C exposure (Table 5). Such high DEPS value resulted from ZEA accumulation under drought (MD of Apoatã and Icatu), and, especially, cold and stresses interaction (SD in all genotypes after chilling), due to both the conversion from the pre-existing VIOL and ANT, and *de novo* synthesis of the xanthophyll cycle pool molecules. The large increase of this xanthophyll agrees with its photoprotective role against the excess of excitation energy at photosynthetic apparatus level in coffee exposed to cold [42,45], and high irradiance [91]. Interestingly, contrasting with ZEA accumulation, *VDE2* was largely down-regulated by cold, and stress interaction on *C*. *arabica* genotypes, whereas it was somewhat promoted by drought in Apoatã at 25/20°C and 13/8°C (Table 6). This could reflect differences at the post-transcriptional regulation level between coffee genotypes [92], but did not limit ZEA synthesis, as shown by the high DEPS values in all genotypes under harsh cold conditions, regardless of water availability. This difference between gene expression and the corresponding enzyme activity (also for APX genes in Icatu plants exposed only to cold) underlined the need to combine molecular, morphological, and physiological studies to assess coffee performance under stress, and to provide accurate tolerance markers [78].

ROS scavenging capability might have been enhanced also by phenolic compounds [15,25], contributing to lower lipoperoxidation [26]. That was reflected by the increases of TPC and 5-CQA (one of the major phenolic compounds in coffee leaves) contents under cold conditions in all genotypes, with the highest value observed in Icatu WW plants upon chilling. In a few cases the stress interaction further increased TPC (*e*.*g*. Apoatã SD and Icatu MD plants), and 5-CQA (Icatu MD plants by 18/13°C and 13/8°C).

Finally, our findings raised important issues regarding coffee crop water management, showing that watering in the cold season must be largely avoided, since plant antioxidative defenses (and photosynthetic performance) can benefit with a pre-water shortage, due to an abiotic stress cross-tolerance mechanisms that mitigate cold impacts.

## Conclusions

The single exposure to cold and drought prompted leaf dehydration, and reduced gas exchanges across genotypes, related both to stomatal and mesophyll limitations. Apoatã was the most affected genotype by cold, with aftereffects persisting in A_max_ by the end of the experiment for all water conditions, but even this genotype showed a faster P_n_ recovery and lower MDA values in droughted plants after cold removal. Icatu plants showed a lower impact under stress and a faster and complete photosynthetic recovery, confirming its higher relative cold tolerance than Apoatã. Interestingly, although lipoperoxidation increased under cold (all genotypes), it was greatly reduced by stress interaction, especially in Icatu. In fact, a general increase of antioxidative enzymes activity was observed in response to the single exposure to drought (except CAT in Apoatã and Icatu) and cold, transversally among coffee species. However, stress interaction further promoted these enzymes activity, with Icatu MD and/or SD plants showing to be the most responsive ones along cold exposure. Therefore, drought was a stronger transcription promoter than cold for some genes related to antioxidative enzymes, but the stress interaction led to the largest transcript accumulation (*APXc*, *APXt+s*, *PX4*), reflecting an aclimatory plant response to oxidative conditions triggered by these stress conditions. Such high transcriptional up-regulation was in line with the APX activity rise, especially in Icatu what was likely related to the lower impacts at photosynthetic and membrane levels in this genotype along the experiments. Additionally, regarding non-enzyme antioxidants, only Icatu showed simultaneous TOC and ASC increases due to drought (MD), and cold (13/8°C and/or after chilling), and a positive stress interaction in TOC (MD plants) at the harshest chilling conditions (the latter also in Apoatã). ZEA was moderately promoted by drought (MD) in Apoatã and Icatu, and highly responsive to cold and stress interaction in all genotypes. TPC and 5-CQA followed a similar pattern of ZEA mostly in Apoatã SD and Icatu MD plants.

In summary, these findings results highlighted the key role of the antioxidative system in the response to drought, cold and their interaction in *Coffea* spp. Drought was mostly an enzyme activity promotor, whereas cold enhanced the complementary synthesis of both enzyme and non-enzyme antioxidants, the latter probably related to a higher need of non-enzyme molecules under cold, when enzyme reactions would be quite repressed. Furthermore, an abiotic stress cross-tolerance was found under this stress interaction, reflected in a supplementary reinforcement of antioxidative capability that reduced lipoperoxidation and protected the photosynthetic machinery in droughted plants along cold exposure, and thereafter, clearer in Icatu. Therefore, antioxidative components have the potential to be used as selection markers in breeding programs regarding cold and/or drought stress tolerance. Finally, these findings are relevant to coffee water management. In fact, although many of newly installed coffee areas have irrigation systems, it was shown that watering in the cold season should be largely avoided in order to allow stress cross-tolerance of the coffee plants.

## Supporting information

S1 Fig Scheme detailing the experiment design using Apoatã, Icatu, and Obatã plants. Plants 1.5 years old in 16 L pots where transferred from the greenhouse to the growth chamber where they stay for 3 months under environmental controlled conditions of temperature (25/20°C, day/night), RH (70%), irradiance at the upper third part of plant canopy (750–850 μmolQ m^-2^ s^-1^), photoperiod (12 h), and air [CO_2_] (390 μL L^-1^). Three groups of 15 plants were then gradually exposed to each of the 3 water availability conditions: well-watered (WW), mild drought (MD) and severe drought (SD) under control conditions (25/20 ^o^C, day/night) along two weeks, with another week for stabilize these water availability levels. Thereafter, plants were exposed to 1) a gradual temperature decrease from 25/20°C to 13/8 ^o^C, over 24 days (0.5°C/day), 2) to a 3 days chilling cycle (3x13/4 ^o^C), where 4 ^o^C were applied during the night and in the first 4 h of the morning (with light), followed by a rise up to 13 ^o^C, throughout the rest of the diurnal period, 3) a rewarming period of 7 days (7x Rec Cold), with the first day after chilling at 20/15 ^o^C and the rest at 25/20 ^o^C, 4) followed by a fully rewatering of all plants, which were allowed to recover for another period of 7 days (7x Rec Drought). The entire experiment last for a total of 62 days since the beginning of the setting of water availability levels.
(TIFF)

## Abbreviations

A_max_: photosynthetic capacity
ANT: antheraxanthin
APX: ascorbate peroxidase
ASC: ascorbate
CAT: catalase
CGA: chlorogenic acids
Chl: chlorophyll
CQA: caffeoylquinic acid
DEPS: de-epoxidation state
diCQA: dicaffeoylquinic acids
DHAR: dehydroascorbate reductase
FQA: feruloylquinic acid
GAE: Gallic acid equivalent
GR: glutathione reductase
GSH and GSSG: reduced and oxidized glutathione
H_2_O_2_: hydrogen peroxide
MDA: malondialdehyde
MDHAR: monodehydroascorbate reductase
^1^O_2_: singlet oxygen
O_2_●^-^: superoxide anion radical
●OH: hydroxyl radical
P_n_: net photosynthetic rate
RWC: relative water content
SOD: superoxide dismutase
TOC: α-tocopherol
TPC: Total Phenol Content
VIOL: violaxanthin
ZEA: zeaxanthin
Ψ_w_: leaf water potential
