# Supplementary figures and images for: Stress cross-response of the antioxidative system promoted by superimposed drought and cold conditions in Coffea spp
Source: PLoS One. 2018 Jun 5;13(6):e0198694. doi: 10.1371/journal.pone.0198694 (PMC5988331; doi:10.1371/journal.pone.0198694)

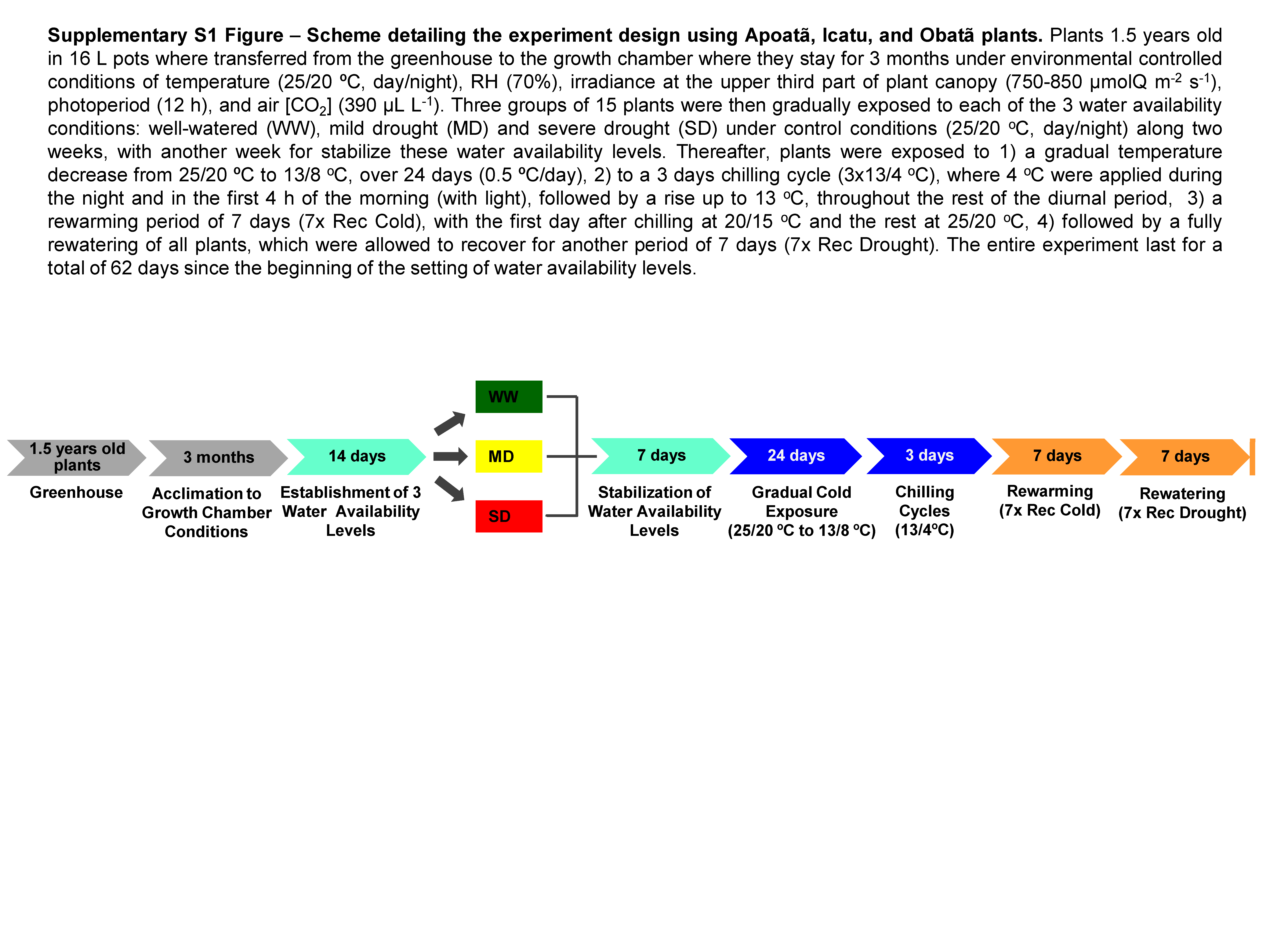

Supplement: S1 Fig — Plants 1.5 years old in 16 L pots where transferred from the greenhouse to the growth chamber where they stay for 3 months under environmental controlled conditions of temperature (25/20°C, day/night), RH (70%), irradiance at the upper third part of plant canopy (750–850 μmolQ m-2 s-1), photoperiod (12 h), and air [CO2] (390 μL L-1). Three groups of 15 plants were then gradually exposed to each of the 3 water availability conditions: well-watered (WW), mild drought (MD) and severe drought (SD) under control conditions (25/20 oC, day/night) along two weeks, with another week for stabilize these water availability levels. Thereafter, plants were exposed to 1) a gradual temperature decrease from 25/20°C to 13/8 oC, over 24 days (0.5°C/day), 2) to a 3 days chilling cycle (3x13/4 oC), where 4 oC were applied during the night and in the first 4 h of the morning (with light), followed by a rise up to 13 oC, throughout the rest of the diurnal period, 3) a rewarming period of 7 days (7x Rec Cold), with the first day after chilling at 20/15 oC and the rest at 25/20 oC, 4) followed by a fully rewatering of all plants, which were allowed to recover for another period of 7 days (7x Rec Drought). The entire experiment last for a total of 62 days since the beginning of the setting of water availability levels. (TIFF) [file pone.0198694.s001.tiff]
